# The Heterogeneous Landscape and Early Evolution of Pathogen-Associated CpG Dinucleotides in SARS-CoV-2

**DOI:** 10.1093/molbev/msab036

**Published:** 2021-02-08

**Authors:** Andrea Di Gioacchino, Petr Šulc, Anastassia V Komarova, Benjamin D Greenbaum, Rémi Monasson, Simona Cocco

**Affiliations:** 1 Laboratoire de Physique de l’Ecole Normale Supérieure, PSL & CNRS UMR8063, Sorbonne Université, Université de Paris, Paris, France; 2 School of Molecular Sciences and Center for Molecular Design and Biomimetics, The Biodesign Institute, Arizona State University, Tempe, AZ, USA; 3 Molecular Genetics of RNA Viruses, Department of Virology, Institut Pasteur, CNRS UMR-3569, Paris, France; 4 Computational Oncology, Department of Epidemiology and Biostatistics, Memorial Sloan Kettering Cancer Center, New York, NY, USA

**Keywords:** ssRNA viruses, SARS-CoV-2, pathogen-associated molecular patterns, pattern recognition receptors, viral host mimicry, CpG motifs, evolution of synonymous mutations

## Abstract

COVID-19 can lead to acute respiratory syndrome, which can be due to dysregulated immune signaling. We analyze the distribution of CpG dinucleotides, a pathogen-associated molecular pattern, in the SARS-CoV-2 genome. We characterize CpG content by a CpG force that accounts for statistical constraints acting on the genome at the nucleotidic and amino acid levels. The CpG force, as the CpG content, is overall low compared with other pathogenic betacoronaviruses; however, it widely fluctuates along the genome, with a particularly low value, comparable with the circulating seasonal HKU1, in the spike coding region and a greater value, comparable with SARS and MERS, in the highly expressed nucleocapside coding region (N ORF), whose transcripts are relatively abundant in the cytoplasm of infected cells and present in the 3′UTRs of all subgenomic RNA. This dual nature of CpG content could confer to SARS-CoV-2 the ability to avoid triggering pattern recognition receptors upon entry, while eliciting a stronger response during replication. We then investigate the evolution of synonymous mutations since the outbreak of the COVID-19 pandemic, finding a signature of CpG loss in regions with a greater CpG force. Sequence motifs preceding the CpG-loss-associated loci in the N ORF match recently identified binding patterns of the zinc finger antiviral protein. Using a model of the viral gene evolution under human host pressure, we find that synonymous mutations seem driven in the SARS-CoV-2 genome, and particularly in the N ORF, by the viral codon bias, the transition–transversion bias, and the pressure to lower CpG content.

## Introduction

When a virus enters a new host, it can present pathogen-associated molecular patterns (PAMPs) that are rarely seen in circulating strains that have adapted to that host’s immune environment over evolutionary timescales. The emergence of SARS-CoV-2, therefore, provides a rare window into innate immune signaling that may be relevant for understanding immune-mediated pathologies of SARS-CoV-2, antiviral treatment strategies, and the evolutionary dynamics of the virus, where evidence for selective pressures on viral features can reflect what defines “self” in its new host. As a case in point, the 1918 influenza pandemic was likely caused by a strain that originated in water fowl and entered the human population after possible evolution in an intermediate host. That viral genome presented CpG dinucleotides within a context and level of density rarely found in the human genome where they are severely underrepresented (Karlin et al. 1994; Karlin and Mrázek 1997; Cheng et al. 2013), particularly in a set of genes coding for the proteins associated with antiviral innate immunity (Greenbaum et al. 2008, 2014). Over the past century, the 1918 H1N1 lineage evolved in a directed manner to lower these motifs and gain UpA motifs, in a way that could not be explained by its usage of amino acid codon bias (cb) (Greenbaum et al. 2008, 2014). It has since been found that these motifs can engage the pattern recognition receptors (PRRs) of the innate immune system (Jimenez-Baranda et al. 2011; Vabret et al. 2017) and directly bind the zinc finger antiviral protein (ZAP) in a CpG-dependent manner (Zhu et al. 2011; Takata et al. 2017; Meagher et al. 2019; Luo et al. 2020). Hence, the interrogation of emergent viruses from this perspective can predict novel host virus interactions.

COVID-19 presents, thus far, a different pathology than that associated with the 1918 H1N1, which was disproportionately fatal in healthy young adults. It has been characterized by a large heterogeneity in the immune response to the virus (Hirano and Masaaki 2020; Mehta et al. 2020; Zhou et al. 2020) and likely dysregulated type-I interferon (IFN) signaling (Kindler and Thiel 2016; Hadjadj et al. 2020; Ragab et al. 2020; Vabret et al. 2020). Various treatments to attenuate inflammatory responses have been proposed and are currently under analysis or being clinically tested (RECOVERYCollaborativeGroup 2020; Xu, Han, et al. 2020). It is therefore essential to quantify pathogen-associated patterns in the SARS-CoV-2 genome for multiple reasons. The first is to better understand the pathways engaged by innate immune system and the specific agonists to help build better antiviral therapies. Another is to better predict the evolution of motif content in synonymous mutations in SARS-CoV-2, as it will help understand the process and timescales of attenuation in humans. Third is to offer a principled approach for optimizing vaccine strategy for designed strains (Amanat and Krammer 2020; Kames et al. 2020) to better reflect human genome features.

In this work, we will use the computational framework developed in Greenbaum et al. (2014) to carry out a study of nonself-associated dinucleotide usage in SARS-CoV-2 genomes. The statistical physics framework is based on the idea of identifying the abundance or scarcity of dinucleotides given their expected usage based on host features. It generalizes the standard dinucleotide relative abundance introduced in Karlin and Mrázek (1997), as it can easily incorporate constraints in coding regions coming from amino acid content and codon usage. The outcomes of the approach are selective forces (Greenbaum et al. 2014) that characterize the deviations with respect to the number of dinucleotides which is statistically expected under a set of various constraints. Such formalism has further been applied to identify noncoding RNA from repetitive elements in the human genome expressed in cancer that engage PRRs (Tanne et al. 2015), to characterize the CpG evolution through synonymous mutations in H1N1 (Greenbaum et al. 2014) and to characterize local and nonlocal forces on dinucleotides across RNA viruses (Vabret et al. 2017).

We perform an analysis of the landscape of CpG motifs and associated selective forces in SARS-CoV-2 in comparison with other ssRNA viruses and other genomes in the coronavirus family in order to understand specific PAMP features in the new SARS-CoV-2 strains. We also focus on the heterogeneity of CpG motif usage along the SARS-CoV-2 genome. Finally, we use a model of viral genome evolution under human host pressure given by the CpG force to study synonymous mutations, and in particular those which change CpG content, observed since the SARS-CoV-2 entered the human population, and study sequence motifs preceding CpG loss loci. The model is used to score all possible synonymous mutations from an ancestral sequence sampled in Wuhan at the beginning of the COVID-19 pandemic (GISAID ID: EPI_ISL_406798) and is validated on single-nucleotide variants observed in the sequence data collected so far. This approach points out at hotspots where new mutations will likely attenuate the virus, while evolving in contact with the human host.

## Results

### Definition of Coding and Noncoding CpG Forces

**Fig. 1.**
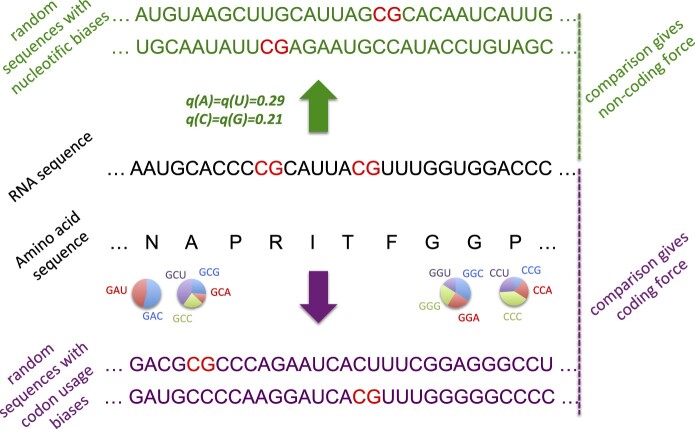
Schematic definitions of CpG noncoding and coding forces. The viral RNA sequence to be analyzed is shown in black, with CpG dinucleotide motifs in red. The force is computed by comparing the occurrences number of CpG with ensembles of random sequences fulfilling some of the constraints acting on the natural sequence, see Materials and Methods for details. Top, “noncoding” framework, in green: sequences of the same lengths can be generated by randomly drawing nucleotides according to prescribed frequencies, here taken from the human genome. Bottom: When the sequence under consideration codes for a protein (sequence of amino acids in black letters), random sequences (violet letters) can be generated in a “coding” framework as follows. For each amino acid, a licit codon is randomly chosen according to prescribed codon usage (here, computed from the coding regions in the human genome). Notice that the above computational frameworks are not restricted to CpG and can be applied to other dinucleotidic motifs.

To characterize CpG dinucleotide usage on SARS-CoV-2 genome, we have computed the CpG forces following the approach introduced in Greenbaum et al. (2014) and described in Materials and Methods. CpG forces are intensive parameters that characterize the abundance or scarcity of CpG dinucleotides in a DNA or RNA sequence with respect to their expected usage relative to a reference nucleotide distribution. We propose two frameworks to define these reference distribution, schematically represented in figure 1. In the noncoding framework, nucleotides are randomly drawn according to a fixed nucleotide bias, whereas in the coding framework, the amino acid sequence is fixed, and the cb defines the reference distribution.

#### Noncoding Forces

The CpG noncoding force relative to the sequence nucleotide bias essentially captures the same information as the relative abundance index, $I=\frac{q(CG)}{q(C)q(G)}$, where *q*(*CG*), *q*(*C*), and *q*(*G*) are, respectively, the frequencies of CpG dinucleotides and of *C*, *G* nucleotides in the sequence (Karlin and Mrázek 1997). The CpG noncoding force is well approximated by the logarithm of the relative abundance index: f≈ log I (see supplementary section SI.3 and fig. SI.13, Supplementary Material online). Positive and negative forces correspond therefore to, respectively, excess and scarcity of dinucleotides with respect to their expected occurrences determined by the nucleotide bias.

Table 1 show that the CpG noncoding forces for human coding (Greenbaum et al. 2014) and noncoding RNA (Tanne et al. 2015) (relative to the human nucleotide bias) are negative, and particularly low for type-I IFN transcripts involved in the innate immune system (Greenbaum et al. 2014), confirming that CpG motifs are overall scarce in the human genome (Karlin et al. 1994; Karlin and Mrázek 1997; Cheng et al. 2013; Greenbaum et al. 2014). As shown in table 1 strongly pathogenic viruses in humans, such as Ebola, the Spanish flu H1N1 (1918) and the avian flu H5N1 (2005), are characterized by large CpG forces compared with the average force on human RNAs. The CpG force value can then be used as an indicator for the propensity of a viral sequence to be sensed by PRRs as nonself and engage the human innate immune response (Greenbaum et al. 2014; Tanne et al. 2015; Vabret et al. 2017). A comparative analysis for noncoding force in the *Coronaviridae* family will be discussed in the following Sections.

**Table 1.** Global Noncoding CpG Forces for Some ssRNA Viruses, Compared with Human RNAs.

	Noncoding CpG Force (±SD)
Ebola 2015	−0.52
H5N1 2005	−0.57 (±0.27)
H1N1 1918	−0.65 (±0.19)
Human coding RNA	−0.66 (±0.95)
H1N1 2009	−0.78 (±0.23)
Human noncoding RNA	−1.17 (±1.42)
Influenza B virus	−1.19 (±0.34)
HIV-1 2020	−1.57
Type I IFNs	−2.07 (±0.83)

Note.—The distribution of forces are computed from all genomic segments and their averages and standard deviations (SD) are given (with segment contribution weighted by segment length). All forces are computed with respect to human nucleotide bias. Data used: human cDNA and ncRNA as annotated in HG38 assembly, transcripts coding for type I IFN’s genes as annotated in HG38. Viral ssRNAs were obtained from NCBI (Wheeler et al. 2007) Virus database (strains used: H5N1: A/Anhui/1/2005, H1N1: A/Aalborg/INS132/2009 and A/Brevig Mission/1/1918, Ebola: COD/1995/Kikwit-9510623, Influenza B virus: B/Massachusetts/07/2020, HIV-1: HK_JIDLNBL_S003).

#### Coding Forces

The CpG coding force is based on the comparison of CpG occurrences in a coding RNA (or DNA) sequence and random synonymous sequences (associated to the same amino acids) drawn according to prescribed codon usage, see figure 1. The computation of coding forces relative to the human codon usage for SARS-CoV-2 will be discussed in the following sections, and it will be used to characterize the evolution of SARS-CoV-2 sequences through synonymous mutations, under the pressure of the human host. To allow for easy comparison and later access, we list in table 2 all CpG coding forces for the *Coronaviridae* family as well as their noncoding counterparts.

**Table 2.** Global Noncoding and Coding CpG Forces for *Coronaviridae* Family Viruses.

	CpG Force (±SD)	
	Noncoding	Coding
MERS	−0.59	−1.13
SARS	−0.82	−1.38
SARS-CoV-2	−1.10	−1.71
hCoVs (229E, HKU1, OC43, NL63)	−1.17 (±0.19)	−1.79 (±0.18)

Note.—All forces are computed with respect to human nucleotide (noncoding forces) or codon bias (coding forces).

### The Landscape of CpG Forces in SARS-CoV-2 Is Strongly Heterogeneous

**Fig. 2.**
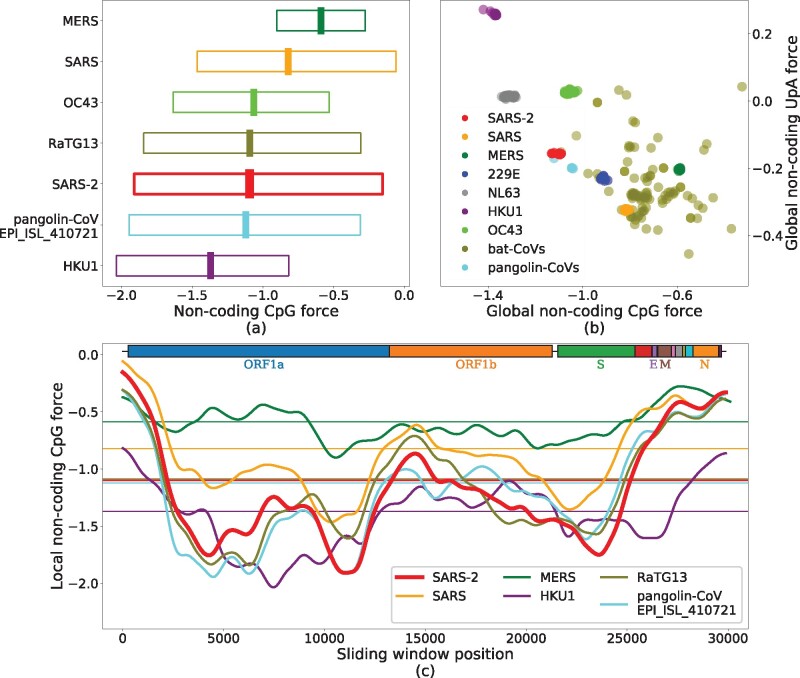
CpG and UpA noncoding forces and local fluctuations in the genomes of the *Coronaviridae* family. (*a*) Noncoding forces on CpG dinucleotides for SARS-CoV-2 and other coronaviruses. The central thick lines show global forces over the whole genomes, whereas bars span from the minimal to the maximal values of the local forces computed on sliding windows along the sequence of 3 kb (narrowed up to 1.5 kb at the edges), smoothed through a Gaussian average. (*b*) UpA versus CpG global forces. Species are well clustered due to the large similarity of the sampled sequences, except for viruses circulating in bats composed of several diverse strains. Notice the anticorrelation between CpG and UpA forces (Pearson r=−0.69, with a *P* value of $≃2\times 10^{-77}$, and $r^{2}=0.48$). (*c*) Local CpG force analysis in sliding windows of 3 kb along the genome for some coronaviruses, smoothed through a Gaussian average; horizontal lines correspond to the global forces shown in panel (*b*). Boxes on top of the panel show protein-coding domains. The force is highly variable along the genome, with much larger values in certain regions (such as the N ORF) than in others (e.g., S ORF). The maximum value of the local CpG force hints at the similarity of SARS-CoV-2 with the most pathogenic viruses (table 2). Data from VIPR (Pickett et al. 2012) and GISAID (Elbe and Buckland-Merrett 2017), see Materials and Methods and supplementary section SI.1, Supplementary Material online, for details on data analysis.

We first computed the global noncoding force on CpG dinucleotides for SARS-CoV-2, a variety of other ssRNA viruses, and other viruses from *Coronaviridae* family affecting humans or other mammals (fig. 2*a*).

The value ≃−1.1 of the global noncoding force for SARS-CoV-2 is comparable with the one for human noncoding RNA and lower than other strongly pathogenic viruses in humans, such as H1N1, H5N1, and Ebola (table 1). Among the coronaviruses (fig. 2*a* and table 2), MERS shows the highest CpG force followed by SARS, whereas some bat coronaviruses have even stronger CpG force. SARS-CoV-2 is among the viruses with lower global CpG force; its value is median among the hCoV that circulates in humans with less pathogenicity, between HKU1 with a smaller CpG force and OC43 with a larger one (supplementary fig. SI.4, Supplementary Material online, for a comparison with other hCoVs). The absence of a straightforward correlation between global CpG force and the pathology of a coronavirus in human calls for a finer, local analysis of CpG forces we report below.

Figure 2*b* compares the forces (comparisons based on dinucleotide number rather than force give qualitatively similar results, see supplementary figure SI.2, Supplementary Material online) acting on CpG and UpA motifs within the *Coronaviridae* family, with a particular emphasis on the genera *Alphacoronavirus* and *Betacoronavirus*, and on those viruses which infect humans (Song et al. 2019); for other dinucleotides, see supplementary figure SI.1, Supplementary Material online. We observe an anticorrelation between UpA and CpG forces (correlation coefficient *R*-squared $r^{2}=0.48$). UpA is the CpG complementary motif corresponding to the two nucleotidic substitutions more likely to occur in terms of mutations, as transitions have larger probability with respect to transversions and are less likely to result in amino acid substitutions. Such anticorrelations are not observed with motifs that are one mutation away from CpG ($r^{2}=0.2$ for CpA vs. CpG and $r^{2}=0.01$ for UpG vs. CpG, supplementary fig. SI.3, Supplementary Material online).

To go beyond the global analysis, we study the local noncoding forces acting on CpG in fixed-length windows along the genome. Results for SARS, MERS, SARS-CoV-2, hCoV-HKU1, and two representative sequences of bat and pangolin coronaviruses, chosen for their closeness to SARS-CoV-2, are reported in figure 2*c*. SARS-CoV-2 shows a strong heterogeneity of CpG forces along its genome: in some genomic regions, especially at the 5′ and 3′ extremities, SARS-CoV-2, SARS, and MERS (together with the bat and pangolin viruses) have a peak in CpG forces, which is absent in the hCoV-HKU1 (as well as in the other hCoVs, see supplementary fig. SI.4, Supplementary Material online). The heterogeneous CpG content in SARS-CoV-2 has been also pointed out in Digard et al. (2020).

The high CpG forces at the extremities could have an important effect on the activation of the immune response via sensing, as the life cycle of the virus is such that the initial and final part of the genome are those involved in the subgenomic transcription needed for viral replication (Lai and Cavanagh 1997; Kim et al. 2020). During the infection, many more RNA fragments from these regions are present in the cytoplasm than from the other parts of the viral genome. Consequently, despite the relatively low CpG content of SARS-CoV-2 compared with other coronaviruses, there can be high concentrations of CpG-rich RNA due to the higher transcription of these regions.

The similarity between the high values of the maximum local forces of SARS-CoV-2 and those of SARS, bat and pangolin coronaviruses shown in figure 2*a* confirm this pattern: MERS and SARS, viruses that are likely less well adapted to a human host, have the highest local peaks in CpG content, followed by SARS-CoV-2 and then by seasonal strains that circulate in humans. It is interesting to notice that high and very high levels of proinflammatory cytokines/chemokines (such as IL-6 and TNF-*α*) have been observed in, respectively, SARS and MERS and, at times, SARS-CoV-2 infection (Zhou et al. 2014; Fajgenbaum and June 2020; Hirano and Masaaki 2020; Vabret et al. 2020). These results are qualitatively corroborated by the simpler analysis of CpG motif density (supplementary fig. SI.2, Supplementary Material online).

### Forces Acting on Coding Regions Widely Vary across Structural Proteins

We now restrict our analysis to the coding regions of SARS-CoV-2 and, in particular, on two structural proteins, N (nucleocapside) and S (spike) (Amanat and Krammer 2020; Hoffmann et al. 2020; Xu, Chen, et al. 2020). As stressed before, the computation of the force in such coding regions accounts for the extra constraints on the amino acid content and takes the human codon bias as reference background.

**Fig. 3.**
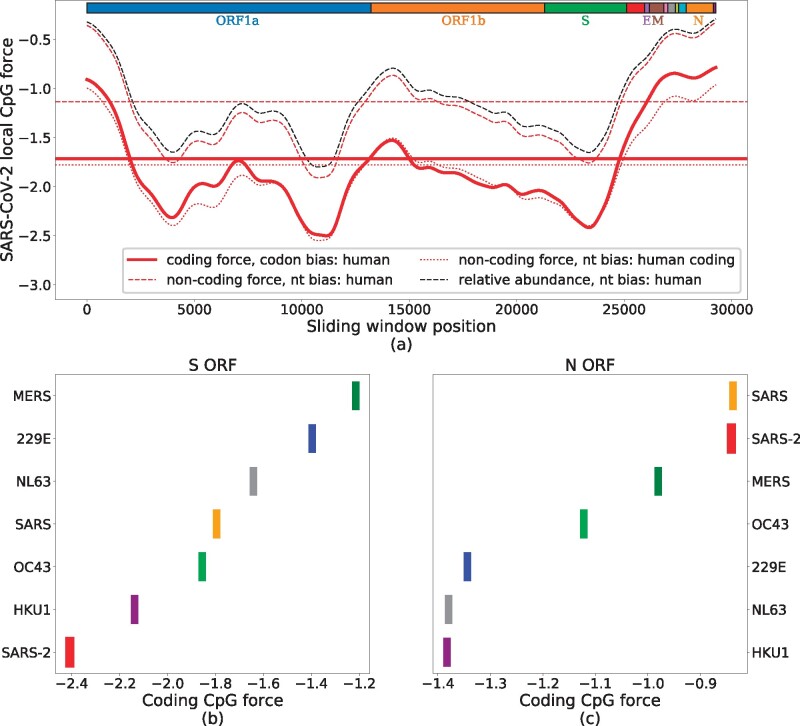
CpG local coding forces on SARS-CoV-2 coding regions. (*a*) Local forces, over sliding windows of 3 kb (narrowed up to 1.5 kb at the edges), in the coding regions of SARS-CoV-2 (preprocessed to ensure the correct reading frame). Full, thick red curve shows coding forces. The dashed and dotted lines show noncoding forces obtained by using nucleotide frequencies computed from, respectively, all human genome (including noncoding RNA and coding RNA) and only coding human RNAs. Horizontal lines locate global forces. The black dashed line shows the relative abundance of CpG computed on the same sliding windows, with the same nucleotide frequency used for the dashed red line. Boxes on top of the panel show protein-coding domains. (*b*, *c*) Global forces for structural proteins (S and N) in the *Coronaviridae* family. These values were averaged on 4–20 sequences from VIPR (Pickett et al. 2012) and GISAID (Elbe and Buckland-Merrett 2017), see Materials and Methods and supplementary section SI.1, Supplementary Material online, for details on data analysis.

The landscape of coding CpG forces with respect to the human codon bias is shown, restricted to the coding regions of SARS-CoV-2 genome, in figure 3*a*, and compared with the noncoding forces from figure 2, with respect to the human nucleotide bias (dashed red lines). The two curves are similar apart from a global shift toward lower forces for the coding forces. Notice that this shift essentially vanishes if the noncoding force is computed with respect to the nucleotide bias computed on human coding RNAs only (Howe and Song 2013; fig. 3*a*).

Apart from this global shift, the strongly heterogeneous landscape of the CpG coding forces along the SARS-CoV-2 genome does not substantially differ from the findings of figure 2*c*. In particular, the peak of high CpG density and force is still present at the 5′ and the 3′ ends of the genome, including the N ORF, the envelope E ORF, and membrane glycoprotein M ORF regions. In the S ORF region, the coding CpG force remains small. Detailed results for the S and N ORFs are shown in, respectively, figures 3*b* and 3*c*. These structural proteins are present and quite similar across the *Coronaviridae* family and allow us to compare several strains of coronaviruses. In the S ORF, SARS-CoV-2 shows the lowest global CpG force among the human-infecting betacoronaviruses, see figure 3*c*. The CpG force is much higher for protein N in SARS-CoV-2, immediately below the level of SARS and above that of MERS, see comparison with human-infecting members of the *Coronaviridae* family presented in figure 3*b*. The comparative analysis of forces in the E ORF (supplementary fig. SI.5*b*, Supplementary Material online) gives results similar to the N ORF, whereas smaller differences in CpG force among coronaviruses that circulate in humans are observed for the M ORF (supplementary fig. SI.5*c*, Supplementary Material online).

### Force-Based Model Accounts for Early Evolution of Synonymous CpG-Related Mutations in SARS-CoV-2

We now assess the ability of our CpG force model to predict biases in the synonymous mutations already detectable across the few months of evolution following the first sequencing of SARS-CoV-2 (data from GISAID; Elbe and Buckland-Merrett 2017; Wuhan ancestral strain has GISAID ID: EPI_ISL_406798, collected in Wuhan on December 26, 2019; last updated sequence September 29, 2020; see Materials and Methods). Barring confounding effects, we expect that high-force regions, such as N ORF, will be driven by the host immune system pressure toward a lower number of CpG motifs. Other regions, such as S ORF, already have low CpG content and would feel no pressure to decrease the CpG content, so random mutations would likely leave their CpG number unaffected or increase it. We define in the following synonymous-single nucleotide variants (syn-SNV) as nucleotide synonymous substitution with respect to the Wuhan ancestral strain, observed at least in five collected sequences (0.01% of the sample) (such cutoff removes very rare mutations which may be due to sequencing errors).

**Fig. 4.**
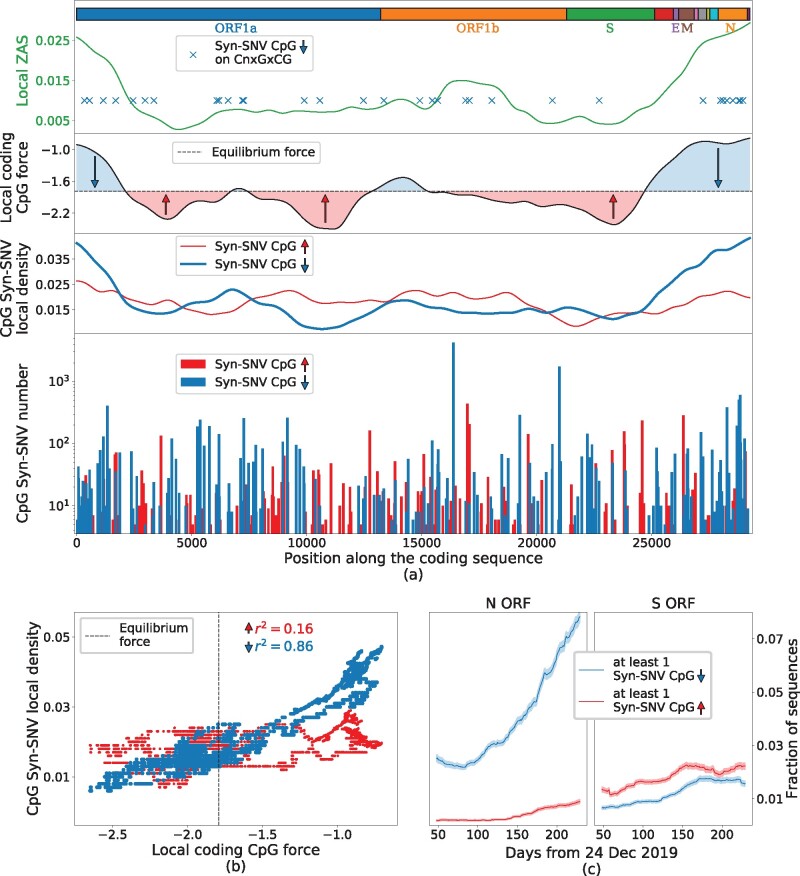
Analysis of synonymous mutations in the early evolution (up to October 2020) of SARS-CoV-2 genome compared with CpG local forces and ZAP-binding motifs. (*a*) Bottom: Counts of syn-SNV that increased (red) and decreased (blue) the CpG content. Middle: local density of syn-SNV increasing (red) and decreasing (blue) CpG averaged on sliding windows of 3 kb and with a Gaussian smoothing; black line: local-coding CpG force with the same sliding average and smoothing; dashed black line: putative equilibrium force (−1.79) for SARS-CoV-2-coding regions. The area between the local CpG force and the equilibrium CpG force is filled in blue/red for local CpG force larger/smaller than the equilibrium one. Upper subpanel: the local ZAS, computed on sliding windows of 3 kb; blue crosses mark SNV removing CpG motifs in a CnxGxCG patterns. Boxes on top of the panel show protein-coding domains. (*b*) Scatter plot of the local CpG force (black curve in panel *a*, without smoothing procedure) versus the local density of CpG decreasing (blue points) or increasing (red points) syn-SNV. Dashed vertical line: putative equilibrium force. (*c*) Fraction of sequences in the data with at least one syn-SNV decreasing (blue curve) or increasing (red curve) the CpG content in the N ORF (left) and S ORF (right), as function of time. To reduce noise, for each point, we considered all the sequences collected in a temporal sliding window of 100 days centered on the point. Data from GISAID (Elbe and Buckland-Merrett 2017), see Materials and Methods (last update October 05, 2020) for details on data analysis. Ancestral genome: GISAID ID: EPI_ISL_406798 (Wuhan, December 26, 2019).

Figure 4*a* (bottom and middle panels) shows that many syn-SNV that decrease the number of CpG are located at the 5′ and 3′ ends of the sequence, in correspondence with the high peaks in CpG local force, notably in the N ORF region and at the 5′ extremity of the genome. Conversely, syn-SNV that increase the number of CpG are more uniformly spread along the sequence. The behaviors of the local CpG force and of the local density of CpG-decreasing syn-SNV, computed on the same sliding windows along the sequence, show strong similarities, see middle panels in figure 4*a*.

To better explain this CpG mutational trend along the sequence, we define a putative equilibrium CpG force of the SARS-CoV-2 genome in human host, as the average CpG force of hCoVs in table 2: hCoVs have long been circulated in humans and are, therefore, supposed to be close to equilibrium with their host. Other choices for equilibrium force will be discussed later. Regions with a CpG force much larger than the equilibrium one are predicted to be under strong evolutionary pressure to decrease their CpG content. This prediction is confirmed by the fact that CpG-decreasing syn-SNV are much more frequent than CpG-increasing ones, see figure 4*a*, middle. Conversely, in regions with forces slightly smaller than the equilibrium force value, the presence of a small evolutionary pressure to increase CpG is confirmed by the fact that CpG-increasing syn-SNV are slightly more frequent than CpG-decreasing ones. The scatter plot of the local forces and densities of CpG-increasing (blue) and -decreasing (red) syn-SNV along the SARS-CoV-2 Wuhan ancestral strain is shown in figure 4*b*. The correlation coefficient is much larger for CpG-decreasing syn-SNV ($r^{2}=0.85$) than for CpG-increasing mutations ($r^{2}=0.16$). The two scatter plots cross at a local CpG force f≃−1.8±0.2, very close to the equilibrium force, $f_{\text{eq}}=-1.79$. This result supports our choice of the equilibrium force. The global force of SARS-CoV-2 (f=−1.71) is also compatible with this crossing point. On the contrary, other possible choices for the equilibrium force, such as the coding force computed on human type-I IFNs, f=−2.89 would not match the crossing point. The results above suggest to introduce, as will be done in the following, the CpG drive defined as the difference between the CpG local force and the equilibrium CpG force. Table 3 complements figure 4 with a detailed description of CpG-decreasing and -increasing syn-SNV along the ORFs and the 5′ and 3′ untranslated regions (UTRs) of SARS-CoV-2 genome. The regions with high negative CpG drive have a large density of CpG removing mutations, see for instance, 5′UTR, 3′UTR, N ORF, and M ORF. Importantly, syn-SNV are in many loci across the sequence (fig. 4*a*) and taking into account syn-SNV counts in the sequence data or considering unique syn-SNV does not qualitatively affect our conclusions. Focusing on N ORF, remarkably 21% (47% with count) of syn-SNV remove a CpG motif. Such percentage represents a fraction of 75% (94% with counts), among the total number of syn-SNV affecting a CpG. On the opposite, the regions with a small-amplitude, negative or positive, drive such as ORF1a, ORF1b, and S ORF have a low density of CpG-affecting mutations; among the syn-SNV affecting CpG motifs the percentage for syn-SNV adding a CpG motif or removing a CpG motif are comparable. For S ORF, having the largest positive drive, the large majority of synonymous variants, 85% (92% with counts), leaves the CpG content unchanged with only few, 7% (4% with counts), syn-SNV affecting a CpG motif. Among syn-SNV affecting CpG, a slight predominance of CpG increasing syn-SNV is observed with 53% (56% with counts) CpG increasing against 47% (44% with counts) CpG decreasing syn-SNV. Last of all, figure 4*c* shows that, in N ORF, a rapid accumulation of CpG removing syn-SNV is observed in the sampled sequences as a function of the delay between the time of collection and the beginning of the COVID-19 pandemic. This increase is much steeper that the gradual rise of syn-SNV increasing CpG occurrences. In the S region, on the contrary, a gradual rise of syn-SNV is observed both for CpG-increasing and -decreasing mutations, with a slight predominance of CpG-increasing ones. A similar analysis of the CpG force evolution in the sequence sample (supplementary fig. SI.10, Supplementary Material online) does not show any significant changes in the small time elapsed up to now.

**Table 3.** CpG Drive and Analysis of syn-SNV Changing CpG along the SARS-CoV-2 Genome.

					CpG↓SNV			CpG↑SNV		
	*L*	CpG	CpG Drive	SNV	Tot	/SNV (%)	/CpGSNV (%)	Tot	/SNV (%)	/CpGSNV (%)
3′UTR	162	5	−0.87	81	18	22	90	2	2	10
			With counts	6,020	1,597	27	99	20	0.3	1
5′UTR	211	13	−1.67	56	19	34	95	1	2	5
			With counts	48,806	47,446	97	99	328	1	1
N	1,260	39	−0.95	115	24	21	75	8	7	25
			With counts	4,745	2,239	47	94	146	3	6
M	669	20	−1.00	58	12	21	86	2	4	14
			With counts	5,508	245	5	45	302	6	55
ORF10	117	5	−2.01	6	2	33	100	0	0	0
			With counts	233	14	60	100	0	0	0
ORF7a	366	7	−0.61	23	5	22	56	4	17	44
			With counts	544	243	45	88	33	6	12
ORF8	366	8	−0.82	25	5	20	63	3	12	38
			With counts	2,146	107	5	86	17	1	14
ORF3a	828	17	−0.62	54	10	19	67	5	9	33
			With counts	1,530	244	16	87	36	2	13
ORF1a	13,203	160	0.05	848	86	10	52	81	10	49
			With counts	79,937	3,930	5	72	1,560	2	28
ORF1b	8,088	115	0.003	432	47	11	48	52	12	53
			With counts	33,788	7,342	22	84	1,434	4	26
E	228	11	−1.84	10	1	10	100%	0	0	0
			With counts	311	69	22	100	0	0	0
S	3,822	29	0.61	223	16	7	47	18	8	53
			With counts	14,540	571	4	44	729	5	56
ORF6	186	1	0.18	10	0	0	0	2	20	100
			With counts	275	0	0	0	40	15	100
ORF7b	132	1	−0.28	9	0	0	0	2	22	100
			With counts	406	0	0	0	14	4	100

Note.—The table gives, for all the ORFs and the 5′ and 3′UTRs of SARS-CoV-2 ancestral genome, the length of the region (*L*), the number of CpG motifs (CpG), the CpG drive $\left(f_{\text{eq}}-f\right)$, the syn-SNV, and the total numbers and percentages of syn-SNV removing a CpG motif (CG↓) or adding it (CG↑), with respect to total number of syn-SNV (/SNV) or to the total number of syn-SNV affecting CpG (/CpGSNV). For the noncoding 5′ and 3′UTRs, all SNV are taken into account with no restriction to syn-SNV and the noncoding forces are used; the equilibrium force is −1.16 (and not −1.79 as for ORFs) releasing such constraint. UTRs and ORFs and are sorted according to the density of CpG removing SNV (CG↓ SNV/L). The regions underlined are the most reliable for statistical analysis as they present at least 20 syn-SNV. Numbers and percentages of SNV are given with and without taking into account SNV counts. Data from GISAID (Elbe and Buckland-Merrett 2017), see Materials and Methods for details on data analysis (last update October 05, 2020). Ancestral genome GISAID ID: EPI_ISL_406798. SNV with <5 counts are excluded from the data. ROC: Receiver Operating Characteristic; AUROC: Area Under the Receiver Operating Characteristic.

### Analysis of Synonymous Mutations in N ORF Suggests Implication of ZAP in Progressive Loss of CpG

We have then studied the nucleotidic patterns preceding, along the viral sequence, the CpG dinucleotides lost in syn-SNV encountered so far. In N ORF, the ORF with largest density of CpG decreasing syn-SNV as shown in table 3, 24 syn-SNV removing one CpG have been found. The nucleotide motifs preceding these loci are listed in the top 19 lines of table 4 (for some loci, more than one syn-SNV removed the same CpG), together with their positions along N ORF of SARS-CoV-2 and their number of occurrences in the sequence data. Seven out of 19 of these loci, which represent 71% of total syn-SNV removing a CpG (1,587 out of the 2,239), correspond to a motif of the type CnxGxCG, where nx is a spacer of n nucleotides and were identified as ZAP-binding patterns in (Luo et al. 2020). The binding affinity of ZAP to the motifs was shown to depend on the spacer length, *n*, with top affinity for *n *=* *7 (Luo et al. 2020) (table 4). Notice that 43% (three out of the seven) of the CpG-suppression-related motifs in SARS-CoV-2 correspond to *n *=* *7. Other motifs of the type CnxGcCG are also present in SARS-CoV-2, but their CpG is not lost in sequence data, see last five lines of table 4; the dissociation constants associated to their spacer lengths are on average larger than the ones of the motifs showing CpG loss.

**Table 4.** Analysis of Nucleotidic Motifs Preceding CpG in the N ORF and Their ZAS.

Motif	*n*	Position of CpG	CpG↓ syn-SNV (with counts)	ZAS
**C**AUUG**G**C**CG**	4	905	509	3.03
**C**GGAAU**G**U**CG**	5	953	607	2.19
**C**AUAUU**G**A**CG**	5	1,074	13	2.04
**C**GCAGUGG**G**G**CG**	7	104	54	8.48
**C**UAACAAA**G**A**CG**	7	384	385	8.33
**C**UGGCAAU**G**G**CG**	7	642	9	8.33
**C**GAGGACAA**G**G**CG**	8	213	10	1.71
CCCCGCAUUA**CG**	—	47	35	0.30
AAUAAUACUG**CG**	—	149	5	0.15
CUUGGUUCAC**CG**	—	162	17	0.15
AUGCUGCAAU**CG**	—	471	34	0.15
AGAAGGGAGG**CG**	—	534	6	0.15
CACAAGCUUU**CG**	—	822	194	0.15
UUGCCCCCAG**CG**	—	930	15	0.15
AGCGCUUCAG**CG**	—	938	21	0.30
CAGCGUUCUU**CG**	—	945	45	0.30
GUCACACCUU**CG**	—	980	104	0.15
CCUUCGGGAA**CG**	—	986	55	0.30
CAAGCCUUAC**CG**	—	1,148	121	0.15
**C**GGCA**G**A**CG**	4	829	0	3.18
**C**UACCA**G**A**CG**	5	277	0	2.04
**C**ACGUA**G**U**CG**	5	571	0	2.19
**C**AAAACAAC**G**U**CG**	8	121	0	1.71
**C**GUGGUGGU**G**A**CG**	8	294	0	1.71

Note.—The top seven lines show subsequences of N ORF (of the Wuhan ancestral strain, GISAID ID: EPI_ISL_406798) of the type C*n*xGxCG, where the spacer *n*x (highlighted in red) includes *n* = 4, 5, 7, or 8 nucleotides, for which the CpG dinucleotide was lost in one or more of the syn-SNV. These motifs were shown to be binding patterns for the ZAP protein in (Luo et al. 2020); the dissociation constants were measured for repeated A spacers, with values (in μM) $K_{d}(4)=0.33\pm 0.05,\;K_{d}(5)=0.49\pm 0.10,\;K_{d}(7)=0.12\pm 0.04,\;K_{d}(8)=0.64\pm 0.14$ (Luo et al. 2020). The next 12 lines show the other CpG lost through mutations and their ten preceding nucleotides, which do not correspond to motifs tested in Luo et al. (2020). The last five lines show other subsequences in the N protein corresponding to ZAP-binding motifs (Luo et al. 2020), but for which no loss of CpG is observed in the sequence data. The column ZAS gives the score associated to the subsequence considered, computed from the above dissociation constants (see Materials and Methods for technical details). Data from GISAID (Elbe and Buckland-Merrett 2017), see Materials and Methods for details on data analysis (last update October 05, 2020).

From the spacer length–dependent-binding affinity given in Luo et al. (2020) (table 4), we have computed a score, which we call ZAP affinity score (ZAS), which is related to the probability of having at least one ZAP bound to such motif (see Materials and Methods for technical details). The ZAS computed in sliding windows across the genome is presented in figure 4*a* (top plot): N ORF is the richest region in motifs of the form CnxGxCG, with the largest ZAS. Our analysis is confirmed in table 5, which reports all Syn-SNV removing CpG following an extended sequence motif. Even if N ORF represents only 4% of the total sequence length, 18% of extended motifs CnxGxCG and 26% (58% with counts) of syn-SNV removing a CpG on an extended motif are on this region. In contrast, only two extended motifs of type CnxGxCG were found in 5′UTR even if many repeated CpG at short interspace were present, see supplementary table SI.1, Supplementary Material online.

**Table 5.** ZAS and Syn-SNV Removing CpG Dinucleotides Preceded by ZAP-Binding Motifs across the SARS-CoV-2 Genome.

		CpGext			CpGext↓SNV	
	*L*	Num	/*L* (%)	ZAS/*L* (%)	Num	/CpG↓SNV (%)
**5′UTR**	211	2	1.4	2	3	16
		With counts			88	0.2
**N**	1,260	12	1.0	4	10	42
		With counts			1,587	71
**ORF7a**	336	2	0.6	3	1	20
		With counts			17	7
**ORF1a**	13,203	25	0.2	1	15	17
		With counts			726	18
**ORF1b**	8,088	16	0.2	1	9	19
		With counts			317	4
**S**	3,822	3	0.1	0.3	1	6
		With counts			25	4
**ORF3a**	828	4	0.5	2	0	0
		With counts			0	0
**M**	669	2	0.3	2	0	0
		With counts			0	0

Note.—The table gives for each region with at least one motif of the form C*n*xGxCG with n=4,5,6,7, or 8 (CpGext) the length *L*, the number of CpGext and their number per unit length, the ZAS for unit length (ZAS/*L*), the number of syn-SNV removing a CpG preceding by an extended motif (CpGext↓SNV), and their fraction with respect to the total number of CpG-decreasing syn-SNV. Additional informations are given in supplementary table SI.2, Supplementary Material online. Numbers and percentage of CpGext↓SNV are given with and without taking into account SNV counts. ORFs are sorted according to the density of CpGext removing SNV (CpGext↓SNV/L). On 5′ and 3′UTRs, there are no synonymous restriction on SNV.

N ORF and M ORF show a similar CpG force (table 3) but have a large difference in CnxGxCG-like motif content, as shown in table 5. Remarkably, when considering the counts, the number of CpG-decreasing mutations occurring in N ORF, out of which 71% are on CnxGxCG-like motifs, is 10-fold more than that occurring in M ORF. These results support the existence of early selection pressure to lower CnxGxCG-like motifs in N ORF, where they are particularly concentrated.

### Model Is Able to Discriminate Observed and Nonobserved Single Nucleotide Variants among Early Synonymous Mutations

Our model can be further used to predict the odds of synonymous mutations, either implying CpG or not, from the ancestral SARS-CoV-2 (GISAID ID: EPI_ISL_406798) sequence. For this purpose, we introduce a synonymous mutation score (SMS), defined in Materials and Methods, whose value expresses how likely a mutation is to appear under the joint actions of the CpG force, the codon bias (we consider here the virus codon bias, calculated on the Wuhan ancestral strain, rather than the human cb, as SARS-CoV-2 is likely not in equilibrium with its host yet. This choice will be justified later), and the transition–transversion bias (ttb) (Jiang and Zhao 2006) (we consider the canonical ratio 4:1 here, see Materials and Methods for details.). For synonymous mutations that do not affect CpG the only mutational driving factors in our model are the cb, and the ttb, which are global drives on the genome. Synonymous mutations changing CpG are additionally driven by the local force to increase or decrease CpG content depending on the CpG mutational drive in the region under consideration (fig. 4*a* and table 3).

**Fig. 5.**
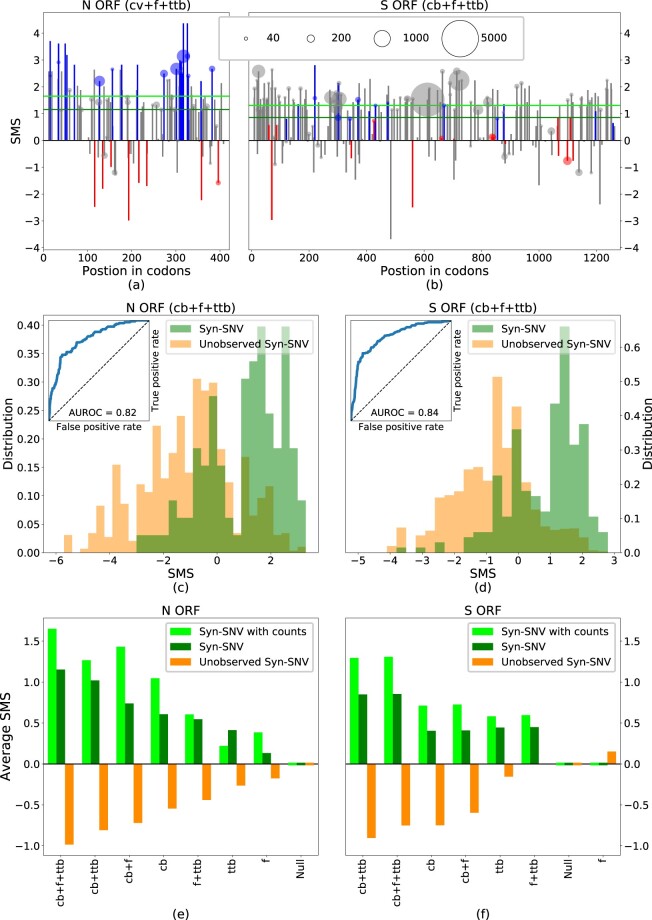
SMS differentiates syn-SNV from unobserved syn-SNV. (*a*, *b*) SMS for syn-SNV with the full model including codon bias (cb), CpG force (*f*), and transition-transversion bias (ttb) in the N and S ORFs. Blue, red, and gray bars denote mutations decreasing, increasing, or leaving unchanged the CpG content. The area of circles, shown on SNV observed more than 20 times in the data set, is proportional to the SNV count. Green, horizontal lines are the average SMS of the syn-SNV with (dark green) and without (light green) counts. (*c*, *d*) Histograms of SMS distribution for observed (green) and unobserved (orange) syn-SNV in the N and S ORFs with the full model (cb + *f* + ttb). The corresponding ROC curve is given as an inset, together with the AUROC. (*e*, *f*) Average SMS for syn-SNV (dark green), syn-SNV with SNV-counts (light green), and for unobserved syn-SNV (orange) computed with the full model and all possible reduced models. In the null model (Null), all synonymous mutations are equiprobable. Models are ranked according to the difference of average SMS for observed and unobserved syn-SNV. Data from GISAID (Elbe and Buckland-Merrett 2017), see Materials and Methods for details on data analysis (last update October 05, 2020). Wuhan ancestral genome has GISAID ID: EPI_ISL_406798. SNV with <5 counts are considered as unobserved syn-SNV.

Figure 5*a* and 5*b* shows SMS along, respectively, the N ORF and S ORF, for all the observed Syn-SNV lowering (blue), increasing (red), or leaving unchanged (gray) CpG content, along with their counts in the sequence sample. In N ORF as in S ORF, the majority of Syn-SNV have high SMS, validating the model predictions. The sign and amplitude of the SMS for CpG-affecting Syn-SNV in figure 5*a* and 5*b* result from the interplay between the virus cb with the CpG drive in the model: the virus cb, computed on the whole genome which is globally low in CpG content, already favors synonymous mutations removing CpG. Due to the cb, both in N ORF and S ORF, Syn-SNV removing CpG tend to have a positive SMS, whereas Syn-SNV adding CpG tend to have a negative SMS (see supplementary fig. SI.12, Supplementary Material online, for SMS computed without CpG drive along N ORF and S ORF). The large and negative CpG drive in N ORF adds to the cb trend. As shown in figure 5*a*, it further raises the SMS of CpG-removing Syn-SNV and further decreases the negative SMS of CpG-adding Syn-SNV, in agreement with the data. The resulting SMS amplitude for CpG-affecting mutations is generally larger than for mutations nonaffecting CpG. On the opposite, in S ORF, as shown in figure 5*b* (see also supplementary fig. SI.12, Supplementary Material online), the positive drive acts against the cb trend, reducing or raising the SMS for, respectively, CpG-decreasing or -increasing Syn-SNVs. In some loci, the CpG drive is strong enough to reverse the sign of the SMS for CpG-increasing Syn-SMS, and the SMS become positive. The resulting SMS amplitude for CpG-affecting mutations is generally smaller than for mutations leaving CpG content unchanged, in agreement with the observation than CpG-affecting Syn-SNV are rare in S ORF.

To make our arguments more quantitative, we tested the ability of our model to discriminate between observed and nonobserved Syn-SNV in sequence data collected so far. For the sake of clarity, nonobserved Syn-SNV refer to the set of possible synonymous mutations that have been not observed so far in the sequence data (or observed very rarely, i.e., with <5 counts). In figure 5*c* and 5*d*, we show that the distribution of SMS for syn-SNV are shifted to higher values compared with its counterpart for nonobserved syn-SNV, both in N ORF and S ORF. Hence, our model is able to statistically discriminate between syn-SNV and nonobserved syn-SNV (ANOVA *F*-test: 1,085 for N and 5,590 for S). Moreover, when ranking all possible synonymous mutations in decreasing order according to their SMS and considering them as true predictions if they have been observed in sampled sequences so far, and thus correspond to an syn-SNV, or false predictions if they have not been observed in the collected sequences, we obtained very good classification performances (AUROC = 0.82 for N and 0.84 for S). As a complementary test we give in supplementary figure SI.8, Supplementary Material online, the positive predicted value as a function of the number of predictions showing that about 85% of the top scored 90 and 150 possible mutations, in N ORF and S ORF, respectively, are syn-SNV.

To identify what ingredient in the model is responsible for correctly distinguishing between observed and nonobserved synonymous mutations, we compare the discriminatory performances of the full model (including the cb, the ttb, and the CpG drive) with all model variations obtained removing one ingredient at the time, up to a null model in which all synonymous mutations are equally likely and all have zero SMS. In figure 5*e* and 5*f*, we compare, for the different models, the average SMS for Syn-SNV, obtained both with and without their counts in the sequence sample (see horizontal light and dark green lines in fig. 5*a* and 5*b*), with the average SMS for the nonobserved syn-SNV. The models are ranked by the difference of average SMS between observed and nonobserved Syn-SNV. As expected, the null model is unable to distinguish between the two sets, assigning vanishing average SMS to both. The gap between the average SMS of observed and nonobserved Syn-SNV progressively increases when introducing back the different ingredients of the model, up to the full one.

We have checked that the choice of the viral codon bias is important for such discriminatory ability. When using the human codon bias instead of the viral one, the model based only on the human codon bias behaves similar to the uniform bias model; the full model is again the best model but with a smaller difference in averages SMS (supplementary fig. SI.6, Supplementary Material online). Classification performances are, on the contrary, unaffected when changing the equilibrium force to the values already discussed, such as the global force SARS-CoV-2 or the average force computed on transcripts encoding human type-I IFNs (supplementary fig. SI.7, Supplementary Material online). It is worth noticing that models based on viral codon bias and ttb alone (without the force) already provide good discriminatory performances in terms of classification of SNV (AUROC and *F* score). This result is expected from the predominance of syn-SNV not affecting CpG occurrences, especially in S ORF. In addition, in regions, such as N ORF, where CpG lowering mutations are frequently observed, the viral codon bias, due to the low global CpG content, favors such synonymous mutations in agreement with figure 5*e* and 5*f*.

Table 6 gives the average SMS and differences, as well as AUROC and ANOVA tests, for all ORFs and 5′ and 3′UTR. We consistently find that the full model is always a good model to describe syn-SNV observed in the early evolution of SARS-CoV-2. There is a clear separation between observed and unobserved syn-SNV average SMS with large AUROC for all the ORFs and UTRs (≥0.68 for all the ORFs and UTRs and ≥0.71 for the regions with a better mutational statistics, with at least 20 syn-SNV) and a large ANOVA *F* value (≥3 for all the ORFs and UTRs and ≥13 among regions with at least 20 syn-SNV); the ORF1a, S ORF, and ORF7b are the only regions for which the average difference between SMS of observed and nonobserved syn-SNV computed with no CpG force is slightly larger. This again suggests that, for the S ORF, syn-SNV are only marginally affecting CpG motifs and are mainly driven by codon bias and ttb.

**Table 6.** Model Performance in Predicting SNV on SARS-CoV-2 UTRs and ORFs.

	CpG Drive	Average SMS					ANOVA *F*-test	
		SNV	u-SNV	Diff	Diff (cb + ttb)	AUROC	*F*	Num items
**3′UTR**	−0.87	0.51	−0.49	1.00	0.73	0.80	101.2	81 + 405
**5′UTR**	−1.67	1.11	−0.37	1.48	0.91	0.84	88.8	56 + 577
**N**	−0.95	1.15	−0.99	2.14	1.83	0.82	153.5	114 + 977
**M**	−1.00	1.29	−0.84	2.13	1.81	0.84	90.8	58 + 557
ORF10	−2.01	2.04	−0.63	2.67	1.89	0.82	9.1	6 + 87
**ORF7a**	−0.61	0.33	−0.87	1.22	1.08	0.71	12.7	23 + 289
**ORF8**	−0.82	0.84	−0.98	1.82	1.62	0.80	33.6	25 + 266
**ORF3a**	−0.62	1.12	−0.90	2.01	1.86	0.84	90.7	54 + 642
**ORF1a**	0.05	0.93	−0.88	1.82	1.83	0.86	1584.4	848 + 9,966
**ORF1a** (no CpG drive)		0.93	−0.89	1.83	—	0.86	1580.3	848 + 9,966
**ORF1b**	0.003	0.78	−0.86	1.63	1.63	0.82	625.0	432 + 6,026
E	−1.84	0.53	−0.64	1.17	0.91	0.68	3.0	10 + 217
**S**	0.61	0.85	−0.75	1.61	1.75	0.84	361.6	223 + 2,924
**S** (no CpG drive)		0.85	−0.90	1.75	—	0.84	375.2	223 + 2,924
ORF6	0.18	0.39	−0.54	0.93	0.91	0.71	5.8	10 + 127
ORF7b	−0.28	0.64	−0.43	1.07	1.11	0.74	5.4	9 + 98
ORF7b (no CpG drive)		0.70	−0.41	1.11	—	0.77	6.1	9 + 98

Note.—The table gives for the SARS-CoV-2 UTRs and ORFs the CpG drive ($f_{\text{eq}}-f$), the average SMS for syn-SNV and unobserved syn-SNV (u-SNV) in the data collected so far (SNV with <5 counts are considered as unobserved), with the full model including cb, CpG drive (*f*), and ttb in the N and S ORFs, the average SMS difference (diff) between the syn-SNV and the unobserved syn-SNV. To assess the role of the force in the model, we also provide difference in averaged SMS for the model not taking into account the CpG drive (diff (cb + ttb)). For ORF1a, S and ORF7b the presence of the CpG drive slightly decreases the average SMS difference, hence we provide the AUROC and ANOVA *F*-test results also with the model without CpG drive (see supplementary table SI.3, Supplementary Material online, for all ORFs and UTRs). All ANOVA *F* values are significant (*P* value <0.05) at exception for E ORF (*P* value = 0.06). The number of items (num items) in the two sets to compute *F* are the syn-SNV + all the possible unobserved syn-SNV. SNV on 5′ and 3′UTRs have not the synonymous restriction.

Remarkably, the ranking of models and their discriminatory performances are essentially the same when taking into account or not the counts of the syn-SNV, even if a net increase in the average syn-SNV SMS score is present for models with the CpG drive in most ORFs and UTRs, and especially in N ORF, when counts are considered (supplementary table SI.4, Supplementary Material online). On the one hand, ignoring counts leads to conservative estimates of the SMS, as mutations that are fixing in the population are not weighted more than less-frequent mutations. On the other hand, SMS based on sequence counts are likely plagued with phylogenetic and sampling biases. We expect that SMS, when properly deriving mutational fitness from phylogeny (Łuksza and Lässig 2014) and correcting for sampling bias, will lie in between the two limit-case SMS discussed above.

## Discussion

The present work reports analysis of dinucleotide motif usage, particularly CpG, in the early evolution of SARS-CoV-2 genomes up to October 2020. First, a comparative analysis with other genomes shows that the overall CpG force, and the associated CpG content are not as large as for highly pathogenic viruses in humans (such as H1N1, H5N1, Ebola, and SARS and MERS in the *Coronaviridae* family). However, the CpG force, when computed locally, displays large fluctuations along the genome. This strong heterogeneity is compatible with viral recombination, in agreement with the hypothesis stated in Andersen et al. (2020). The degree to which this heterogeneity in any way reflects zoonotic origins should be further worked out using phylogenetic analysis. In particular, the segment coding for the Spike protein has a much lower CpG force. The S protein has to bind ACE2 human receptors and TMPRSS2 (Hoffmann et al. 2020; Vabret et al. 2020). A fascinating reason that could explain the low CpG force on this coding region is that it may come (at least in part) from other coronaviruses that better bind human entry receptors (Andersen et al. 2020; MacLean et al. 2020). Other regions, in particular, the initial and final part of the genome, including the 5′ and 3′UTR and N ORF, are characterized by a larger density of CpG motifs (and corresponding CpG force), which are comparable with what is found in SARS and MERS viruses in the *Betacoronavirus* genus. Interestingly, the initial and final part of the genome are implied in the full-genome and subgenomic viral replication. In particular, the coding region of the N protein and its RNA sequence, present in the 3′UTRs of all SARS-CoV-2 subgenomic RNAs, has been shown in Kim et al. (2020) to be the most abundant transcript in the cytoplasm. The high concentration of N transcripts in the cytoplasm could contribute to a dysregulated innate immune response. A mechanism generating different densities of PAMPs being presented to the immune system at different points in the viral life cycle can affect immune recognition and regulation. The precise way this can contribute to immunopathologies associated with COVID-19 and how this is related to the cytokine signaling dysfunction associated with severe cases (Ragab et al. 2020) need further experimental investigation.

The analysis of the evolution of synonymous mutations since the outbreak of COVID-19 shows that mutations lowering the number of CpG have taken place in regions with higher CpG content, at the 5′ and 3′ ends of the sequence, and in particular in the N protein-coding region. The sequence motifs preceding the loci of the CpG removed by mutations match, especially in N ORF, some of the strongly binding patterns of the ZAP protein (Luo et al. 2020). Natural sequence evolution seems to be compatible for protein N with our model, in which synonymous mutations are driven by the virus codon bias and the CpG forces leading to a progressive loss in CpG. These losses are expected to lower the CpG forces, until they reach their equilibrium values in human host, as is seen in hCoV coronaviruses commonly circulating in human population (Abdul-Rasool and Fielding 2010). More data, collected at an unprecedented pace (Pickett et al. 2012; Elbe and Buckland-Merrett 2017; Hadfield et al. 2018), and on a longer evolutionary time are needed to confirm these hypothesis. Since the data collected are likely affected by relevant sampling biases, a more precise analysis of synonymous mutations could be carried out using the available phylogenetic reconstruction of viral evolution (Gonzalez-Reiche et al. 2020). Nevertheless our results seem robust, because they are consistent both considering unique synonymous variants and including their counts. They coherently point to the presence of putative mutational hotspots in the viral evolution. Although the results presented here are preliminary due to the early genomics of this emerging virus, they have been confirmed by incoming sequence data since our first analysis (dated May 5, 2020, see supplementary fig. SI.11, Supplementary Material online), and they point to interesting future directions to identifying the drivers of SARS-CoV-2 evolution and building better antiviral therapies. In this work, we focused on synonymous mutations, but it would interesting to extend our fixed amino acid model for viral evolution to take into account nonsynonymous mutations and to further model transmission and mutation (in the presence of a proofreading mechanism; Denison et al. 2011) processes in SARS-CoV-2 to predict the timescale at which natural evolution driven by host mimicry would bring the virus to an equilibrium with its host (Greenbaum et al. 2008, 2014).

After our work was posted on the *bioRxiv*, Nchioua and colleagues have shown the importance of ZAP in controlling the response against SARS-CoV-2 (Nchioua et al. 2020) by demonstrating that a knockout of this protein increases SARS-CoV-2 replication. The interaction between SARS-CoV-2 and ZAP has also been observed with unbiased methods in another recent work (Flynn et al. 2020). This finding supports our prediction that recognition of SARS-CoV-2 by ZAP imposes a significant fitness cost on the virus, as demonstrated by its early evolution to remove ZAP recognition motifs. Two other recent theoretical works (Wei et al. 2020; Sadykov et al. 2021) corroborate our results showing that at the single nucleotide level, there is a net prevalence of C→U synonymous mutations (the most common nucleotide mutation which may cause a CpG loss) in the early evolution of SARS-CoV-2. Moreover, a recent analysis of the immune profile of patients with moderate and severe disease revealed an association between early, elevated cytokines and worse disease outcomes identifying a maladapted immune response profile associated with severe COVID-19 outcome (Lucas et al. 2020).

## Materials and Methods

### CpG Density Versus Local and Global Forces

Throughout this work, we used CpG forces to quantify the CpG content of a given sequence. Here we want to compare this approach with the simple count of CpG motifs in the sequence. In supplementary figure SI.2, Supplementary Material online, we show that some of our results, such as the large fluctuations of the CpG content across the SARS-CoV-2 genome, are also apparent from a simple motif density analysis. However, this counting strategy is not suitable to make comparisons among viruses of different families, mainly because of the different lengths and usage biases of viral genomes. Moreover, without the statistical framework at the basis of the CpG force, it is very difficult to take into account the many constraints acting on a genetic sequence, notably the constraint on the amino acids that have to be coded for in the sequence.

The force formalism is, therefore, much more flexible and allows us to introduce in a theoretically grounded way the synonymous mutation score, which we used to characterize mutations that are likely to happen. The drawback of such a formalism is the quite large number of extra choices that have to be done, and which can influence the result. These choices are discussed in the following.

### Force Computation

The technique at the core of many of the analyses made here is taken from Greenbaum et al. (2014). Here we briefly review this technique, starting from its noncoding version that takes as reference bias the nucleotide bias and then describing the coding version that takes as reference bias the codon bias at fixed amino acid sequence.

#### Force Computation in Noncoding Case

Given a motif *m* and a sequence $s_{0}=\{s_{1},\ldots ,s_{N}\}$ of length *N*, we consider the ensemble of all sequences with length *N*, which we denote with S, and we suppose the probability of observing *s* out of this ensemble to be

(1) $$p(s)=\frac{1}{Z}\left(\prod_{i=1}^{N}q(s_{i})\right)e^{f_{\text{nc}}N_{m}(s)}.$$

Here, $q(s_{i})$ is the nucleotide bias, that is, the probability of the *i*th nucleotide being *s_i_* (e.g., we always used in this work the human frequency of nucleotides as $q(s_{i})$), *f*_nc_ is the force we want to compute (the subscript nc stands for noncoding), and *N_m_* is the number of times the motif *m* appears in the sequence. *Z* is the normalization constant, that is,

(2) $$Z=\sum_{s\in S}\left(\prod_{i=1}^{N}q(s_{i})\right)e^{f_{\text{nc}}N_{m}(s)}.$$

Therefore, the force *f*_nc_ is a parameter that quantifies the lack (if negative) or abundance (if positive) of occurrences of *m* with respect to the number of occurrences due to the local probabilities $q(s_{i})$. We can fix *f*_nc_ by requiring that the number of motifs in the observed sequence, $N_{m}(s_{0})=n_{0}$, is equal to the average number of motifs computed with the probability equation (1), 〈n〉, that is,

(3) $$\langle n\rangle =\frac{1}{Z}\sum_{s\in S}\left(\prod_{i=1}^{N}q(s_{i})\right)N_{m}(s)e^{f_{\text{nc}}N_{m}(s)}.$$

Notice that this is equivalent to the request that *f*_nc_ is so that probability of having observed *s*_0_ is maximum.

Let us focus now on the specific case of a dinucleotide motif, that is, our motif *m* consists of the pair *ab*, where *a* and *b* are two consecutive nucleotides (e.g., *a *=* C* and *b *=* G* for the CpG motif). In this case, within an approximation discussed in the supplementary section SI.3, Supplementary Material online, the force computed as above turns out to be the logarithm of the relative abundance index, that is,

(4) $$f_{\text{nc}}≃\;\text{log}\;\left(\frac{q(ab)}{q(a)q(b)}\right),$$

where *q*(*ab*) is the number of motifs *ab* divided by the total length of the sequence *N*. In supplementary figure SI.13, Supplementary Material online, we tested the accuracy of this approximation in our specific case. As it is clear from equation (4), the choice of the nucleotide bias *q*(*s*) affects the absolute value of the forces but not the difference between forces computed on different viral genomes using the same reference bias. We have chosen as reference nucleotide bias the human nucleotide bias (computed on all the genome or on the coding DNA only). This choice can be then replaced by any other reference bias (possible choices include the cb computed on the ancestral SARS-CoV-2 sequence or other human Coronavirus viral sequences or the one computed on RNA transcripts of human type-I IFNs, at the core of innate immune response) and will shift the values of the forces without affecting the ranking of the force on different viral sequences, see figure 2 and table 1.

#### Force Computation in the Coding Case

Our technique can be generalized to coding sequences at fixed amino acid sequence and codon bias. In this case, we write each sequence *s* as a series of codons, and its probability is defined as

(5) $$p(s)=\frac{1}{Z}\left(\prod_{i=1}^{N/3}q(c_{i})\right)e^{f_{c}N_{m}(s)},$$

where now the bias takes the form of a codon usage bias, and the normalization constant *Z* changes accordingly into a sum over all possible synonymous sequences. The subscript c stands for coding and differentiates this force from the noncoding one. The force *f*_c_ can be computed, analogously with the procedure for the simpler case, by requiring that the number of motifs observed in *s*_0_ is equal to the statistical average performed with equation (5), as described in detail in Greenbaum et al. (2014). As shown in figure 3*a*, the CpG force at fixed amino acids are roughly comparable with the one at fixed nucleotide bias when computing the nucleotide bias on human coding sequences.

The force computed in the coding (or noncoding case) is an useful tool to determine the content of a given dinucleotide, while taking into account a number of constraints.

### Definition of SMS

We use the ideas discussed above to introduce a model in order to assign a score, which we call SMS, to each possible single-codon synonymous mutation of an ancestral sequence. We consider a system evolving for a small timescale, and a mutation that changes the *i*th codon *c_i_* into a synonymous $c_{i}^{\prime }$. The transition probability, that is, the probability of observing the mutation, for such evolution can be decomposed in the product of two evolution operators: the first $T(N_{\text{CG}}\to N\prime_{\text{CG}})$ representing the change in the number of CpG motifs in the mutated sequence and the second $T(c_{i}\to c\prime_{i})$ representing the gain in mutating the particular codon in position *i*.

The first operator can be computed from the dynamical equation introduced in Greenbaum et al. (2014) for the evolution of the CpG number *N*_CG_ of a sequence under an initial force (Here we drop the subscripts nc and c used in the previous section to identify noncoding and coding forces, since the SMS is defined for a generic force) *f* through an equilibrium force *f*_eq_:

(6) $$\tau \frac{dN_{\text{CG}}}{dt}=(f_{\text{eq}}-f).$$

The equilibrium force can be computed on a viral strain which is supposed to be at equilibrium with the human innate immune system, because it has evolved in the human host since a long time. Equation (6) was used in Greenbaum et al. (2014) to describe the evolution of the CpG number in H1N1, taking as the equilibrium force the one of the Influenza B strain. In analogy with this approach, we take here as *f*_eq_ the average force calculated on coding regions of the seasonal hCoVs (i.e., hCoV-229E, hCoV-NL63, hCoV-HKU1, hCoV-OC43). Other possible choices are discussed below (see also supplementary fig. SI.7, Supplementary Material online). *τ* is a parameter determining the characteristic timescale for synonymous mutations. Based on equation (6), we define the transition operator for CpG number as

(7) $$T(N_{\text{CG}}\to N\prime_{\text{CG}})\propto e^{(f_{\text{eq}}-f)\Delta N_{\text{CG}}},$$

where $\Delta N_{\text{CG}}=N\prime_{\text{CG}}-N_{\text{CG}}$. Notice that for all the synonymous mutations leaving unchanged the CpG number, the above operator is one. The codon mutational operator is

(8) $$T(c_{i}\to c\prime_{i})\propto \left(\frac{q(c_{i}^{\prime })}{q(c_{i})}\right),$$

where $q(c_{i})$ is the frequency of codon *c_i_* from the chosen codon usage bias. Putting together, these two terms allow us to estimate how likely a specific synonymous mutation is to happen. The SMS accompanying a mutation is defined as the logarithm of this quantity,

(9) $$\text{SMS}=(f_{\text{eq}}-f)\Delta N_{\text{CG}}+\text{log}\;\left(\frac{q(c_{i}^{\prime })}{q(c_{i})}\right)\;.$$

To conclude, we remark that different models can be used in the SMS computation, where a model is specified by giving the choice of including or not the force term, the choice of the equilibrium force to be used, the choice of including or not the cb term, and choice of the reference cb to be used.

### Adding transition-transversion bias to SMS

It is well known that transversions (i.e., mutations of purines in pyrimidines and vice versa) are suppressed with respect to transitions (i.e., mutations of purines in purines or pyrimidines in pyrimidines).

We introduce here a simple way to account for transition-transversion bias (ttb) in the model used to assign the SMS. We suppose that a mutation with *n* transversions happens four times less than a mutation with *n −* 1 transversions. This probability ratio, which is a standard value in the literature (Jiang and Zhao 2006), has been recently shown to be close to the observed value for SARS-CoV-2 (Roy et al. 2020). To include that in our model, consider mutating a codon *c* to c′, one of its synonymous codons. Let SMS(c,c′) be the SMS for this event, computed with a given model. We then count the number of transitions, *n*_trn_, and the number of transversions, *n*_trv_, and modify the SMS into SMS′, so that

(10) $$\text{SMS}\prime (c,c\prime )=\text{SMS}(c,c\prime )+n_{\text{trn}}\;\text{log}(2)-n_{\text{trv}}\;\text{log}(2).$$

This choice is motivated by mainly two considerations: 1) in this way, a dynamical model where mutation probabilities are proportional to the exponential of SMS (as the one used to justify the SMS itself) correctly gives a 4-fold probability to a transition than a tranversion (if the two mutations have the same SMS without this new term) and 2) the splitting on the extra term in a positive weight for transitions and a negative weight for transversions ensures that the average SMS before and after adding this term is comparable.

### ZAP Affinity Score

We introduce the ZAS to roughly quantify a priori the likelihood of ZAP biding to a given region of RNA. ZAS is based on the dissociation constants obtained in vitro in Luo et al. (2020). Let us consider the case of a single motif (be it CG, or CnxGxCG, with *n* = 4, 5, 6, 7, or 8), M, with dissociation constant *K*_d_. The association constant is then defined as

(11) $$K_{a}=\frac{\left[\text{ZAP}+M\right]}{\left[M][\text{ZAP}\right]},$$

where [ZAP+M] is the concentration of complexes, [ZAP] and [M] are the concentration of free molecules. Let us denote by ${\left[\text{ZAP}\right]}_{0}$ and ${\left[M\right]}_{0}$ the total concentration of molecules (bound and unbound). If we suppose that only a small part of the available molecules form a complex, that is, more specifically that $K_{a}{\left[\text{ZAP}\right]}_{0}\ll 1$ and $K_{a}{\left[M\right]}_{0}\ll 1$, then $K_{a}{\left[\text{ZAP}\right]}_{0}≃K_{a}[\text{ZAP}]$ is the probability of binding. If we have *n* sites with association constants $K_{a}^{\left(1\right)},\ldots ,K_{a}^{\left(n\right)}$, the probability of observing at least one ZAP bound to the RNA is

(12) $$p=1-\prod_{i=1}^{n}\left(1-K_{a}^{\left(i\right)}[\text{ZAP}]\right)≃[\text{ZAP}]\sum_{i=1}^{n}K_{a}^{\left(i\right)},$$

where we also used that *n* is sufficiently small so that $[\text{ZAP}]\sum_{i=1}^{n}K_{a}^{\left(i\right)}\ll 1$. Finally, ZAS is defined as

(13) $$\text{ZAS}=\sum_{i=1}^{n}K_{a}^{\left(i\right)},$$

that is, p/[ZAP]. Although ZAS itself does not depend on [ZAP], its interpretation (and in particular its connection with the probability of binding) does, as it requires $K_{a}^{\left(i\right)}{\left[\text{ZAP}\right]}_{0}\ll 1,\;K_{a}^{\left(i\right)}{\left[M\right]}_{0}\ll 1$, and ${\left[\text{ZAP}\right]}_{0}\sum_{i=1}^{n}K_{a}^{\left(i\right)}\ll 1$. The $K_{a}^{\left(i\right)}$ used here range from about 10^5^ (for the simple CpG motif) to 10^7^ (for C7xGxCG) mol/L. It is more difficult to estimate ${\left[M\right]}_{0}$ and ${\left[\text{ZAP}\right]}_{0}$ during the infection. However, we hypothesize that these requirements are fulfilled in cells, and that our interpretation in terms of binding probability is acceptable.

### Robustness of Analysis with Respect to Choice of Parameters

We discuss here how force values and SMS scores change by changing model parameters.

Parameters affecting the force values:

- *Nucleotide, codon bias choices*: the most relevant effect due to this choice is a global shift of the force, as we show in figure 3*a* for the noncoding case, which does not change the ranking of forces when comparing different sequences using the same reference bias.
- *Choice of the length of the segment to compute the force*: the force is an intensive parameter. However, here we use the force to quantify the content of CpG motifs, which are quite rare. For this reason, computing forces on small segments can lead to large negative values (the force is −∞ when no CpG motif is present) and to unnatural fluctuations. For this reason, to compute local force, we fix large sliding windows of 3 kb, and we use Gaussian sliding averages to smooth the resulting curves. The effect of Gaussian smoothing and changing sliding window on the force are presented in supplementary figure SI.9, Supplementary Material online.

Parameters affecting the SMS:

- *The codon bias (or nucleotide bias for mutations in 5′ and 3′UTRs)*: it is both present as a reference bias in the force computations for the CpG drive term and directly used as a more generic evolutionary driver for the synonymous mutations. For the computation of forces in the CpG drive, we have used the human codon or nucleotide bias as reference usage, but such choice is actually irrelevant because the drive is a difference of the segment force and the equilibrium one. The choice of bias is, on the opposite, very relevant for the choice of the synonymous mutations driver. Indeed, in figure 5*e* and 5*f*, it is apparent that the virus codon bias alone gives to the model a certain ability to discriminate between observed an unobserved syn-SNV. We tested also the human codon bias which gives bad performances (see supplementary fig. SI.6, Supplementary Material online).
- *Choice of equilibrium force*: this choice is arbitrary to a certain degree. We use as equilibrium force (computed with the human codon bias) −1.79 which is the average coding force hCoVs (229E, NL63, HKU1, OC43), since these viruses are well adapted to the human environment so likely a good equilibrium point for SARS-CoV-2. To check the effects of other choices of equilibrium forces, in supplementary figure SI.7, Supplementary Material online, we performed the same analysis shown in figure 5*e* and 5*f* with other two possible choices of equilibrium forces: the global force of SARS-CoV-2 (−1.71, which is quite low, but slightly higher than the average of the seasonal hCoVs), and the average global force of INF-I transcripts (−2.89 which is much lower than that of seasonal hCoVs), see also tables 1 and 2. Although the SMS assigned to the mutations are in general different, especially when taking into account the counts in syn-SNV, and so the average SMS in figures, the ranking of the various models in terms of average SMS difference (syn-SNV vs. unobserved syn-SNV) is quite robust.
- *Presence of ttb*: we observe that the presence of this term always increases the difference between the average SMS in observed and unobserved synonymous SNV. Two choices are needed to fix this term: the value of the probability ratio of a transition with respect to a transversion (here we considered this ratio to be 4), and the specific way of realizing this bias by adding a bonus/penalty term to transitions and trasnversions. The latter choice is almost irrelevant when considering the differences of average SMS between observed and unobserved SNV.

### Data Analysis and Data Availability

SARS-CoV-2 sequences are taken from GISAID (Elbe and Buckland-Merrett 2017). We downloaded each sequence present in the database on October 05, 2020 (the most recent sequence was collected on September 29, 2020). Before any of our analyses, we discarded all the sequences where one or more nucleotides were wrongly read (other characters than A, C, G, T, U). This left us with 56,045 SARS-CoV-2 sequences. To obtain figure 2, we considered, in addition to the SARS-CoV-2 sequences are taken from GISAID, other *Alphacoronavirus* and *Betacoronavirus* sequences (whole genomes and genes) which have been obtained from VIPR (Pickett et al. 2012). The preprocessing consisted again of discarding all the sequences with one error or more. After this process, we collected 341 SARS, 48 MERS, 20 hCoV-229E, 48 hCoV-NL63, 14 hCoV-HKU1, 124 hCoV-NL63, 166 bat-CoVs, and 5 pangolin-CoVs whole genomes. For figure 2*b*, we used the largest number possible of sequences, up to a maximum of 100. For figure 2*a* (viral sequences) and 2*c*, we chose a single sequence for each species. However, we checked that the result is qualitatively the same if we use other sequences from the same species for human coronaviruses. The curves in figure 2*c* are smoothed through a Gaussian sliding average (on windows of 3 kb, the Gaussian being centered in the window, normalized, with a standard deviation of 300 b). The ancestral SARS-CoV-2 sequence used throughout the work has been collected on 26-12-2019 (ID: EPI_ISL_406798).

In figure 3*a*, the SARS-CoV-2 sequence has been processed to ensure the correct reading frame. Therefore, the ORF1ab gene is read in the standard frame up to the ribosomal shifting site, and it is read in the shifted frame from that site up to the end of the polyprotein. Moreover, the small noncoding parts between successive proteins have been cut, resulting in a loss of 634 nucleotides (including the 5′ and 3′UTR). A Gaussian smoothing has been performed to obtain the plotted CpG forces (as in fig. 2*c*). To produce the bar plots in figure 3*b* and *c*, we collected genes data on VIPR. Then we discarded all the sequences with one or more errors, and we computed for each gene an average of up to 20 different sequences (coming from the same species). For some structural proteins, we did not find 20 different genes but in any case, the standard deviation of the averages of figure 3*b* and 3*c* is smaller than 0.02. In particular, we used 20 sequences of SARS-CoV-2, MERS, hCoV-NL63, hCoV-OC43 proteins, 14 sequences for hCoV-229E, 13 for hCoV-HKU1, and 4 for SARS. More detailed information about the genomes used in this work are given in supplementary section SI.1, Supplementary Material online.

The mutations used for figures 4 and 5 have been collected by extracting ORFs from the SARS-CoV-2 sequence data set and comparing them with the Wuhan ancestral strain. ORFs with mutations too close to the start or end codon are not considered, together with ORFs with insertion/deletion, this filtering procedure leaving us with 48,511 sequences to obtain mutation data. Mutations with <5 counts in different sequences are discarded. All curves in figure 4*a* are smoothed with the same Gaussian average used in figures 2 and 3. Finally, to get the mutation data in 5′ and 3′UTR, we considered the UTRs of the Wuhan ancestral strain, and we compared them with those of other sequences. The number of nucleotides of the Wuhan ancestral considered part of 5′ and 3′UTR for the search for mutations in other sequences is given in table 3. This length is chosen so that a large number of uploaded sequences (about 50,000) have a UTR of the same length or longer. In the UTR analysis, all observed mutations are considered “synonymous.”

The code used to compute coding and noncoding forces is publicly available at https://github.com/adigioacchino/dinucleotide_forces (last accessed February 16, 2021).

## Supplementary Material

Supplementary data are available at *Molecular Biology and Evolution* online.

## Supplementary Material

msab036_Supplementary_Data
