# Supplementary material for: The Heterogeneous Landscape and Early Evolution of Pathogen-Associated CpG Dinucleotides in SARS-CoV-2
Source: Mol Biol Evol. 2021 Feb 8;38(6):2428–45. doi: 10.1093/molbev/msab036 (PMC7928797; doi:10.1093/molbev/msab036)
Supplement: msab036_Supplementary_Data [file msab036_supplementary_data.zip › supplementary_v3.pdf]

The heterogeneous landscape and early evolution of pathogen-associated CpG dinucleotides in SARS-CoV-2

Supplementary Information

Andrea Di Gioacchino, Petr Šulc, Anastassia V. Komarova, Benjamin D. Greenbaum, Rémi Monasson, Simona Cocco

SI.1 Genomes analyzed

Here we report some additional information about the genomes used in this work. The SARS-CoV-2 sequence used in Fig. 2 has GISAID accession ID: EPI_ISL_420793. The other GenBank accession numbers for the specific genomes used in Figs. 2 are: AY427439 (SARS), NC_038294 (MERS), MF542265 (hCoV-229E), JX524171 (hCoV-NL63), KT779555 (hCoV-HKU1) and KF923918 (hCoV-OC43). For these figures, we choose the bat and pangolin sequences as the closest to the SARS-CoV-2 points in Fig. 2b (these two sequences are also known to be very similar to the SARS-CoV-2 genome from previous works [1]). These sequences have GISAID accession IDs EPI_ISL_402131 (bat coronavirus sequence known with the name RaTG13) and EPI_ISL_410721 (pangolin coronavirus sequence collected in 2019 in Guangdong).
The SARS-CoV-2 ancestral sequence which has been collected on 26-12-2019 has GISAID accession ID: EPI_ISL_406798. This sequence has been used as reference in Figs. 3, 4 and 5. For Figs. 3b and 3c we used specific sequences, with the following GenBank accession numbers: MT300186:28249-29508 (SARS-CoV-2), AY291315:28120-29388 (SARS), NC_038294:28565-29800 (MERS) and KT779555:28281-29606 (hCoV-HKU1) for the N protein; MT300186:21538-25359 (SARS-CoV-2), AY291315:21492-25259 (SARS), NC_038294:21455-25516 (MERS) and KT779555:22903-26973 (hCoV-HKU1) for the S protein.

SI.2 Supplementary Figures

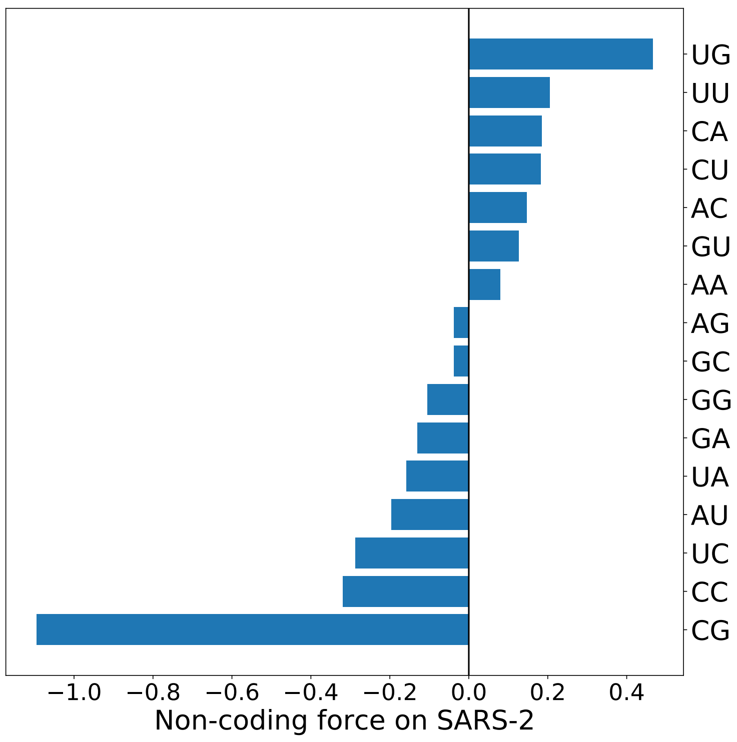

Figure SI.1: All dinucleotide non-coding forces computed on the whole SARS-CoV-2 genome. The CpG motif is the one with the largest non-coding force in absolute value, and the second one is UpG, which is one transition away from CpG.

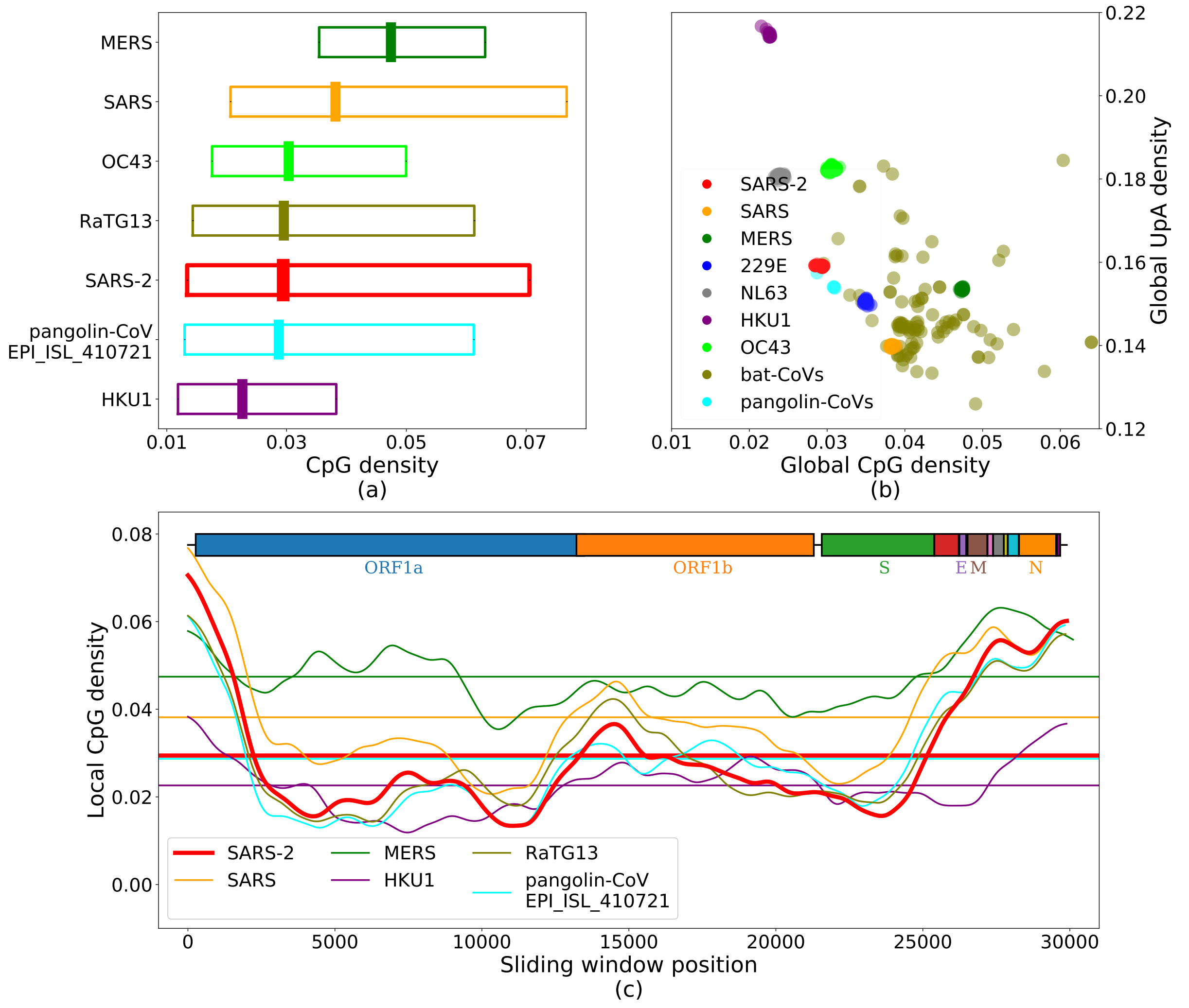

Figure SI.2: The same analysis performed in Fig. 2, but here we used CpG densities (CpG dinucleotides divided by the total number of dinucleotides), instead of CpG forces. The results obtained are qualitatively similar.

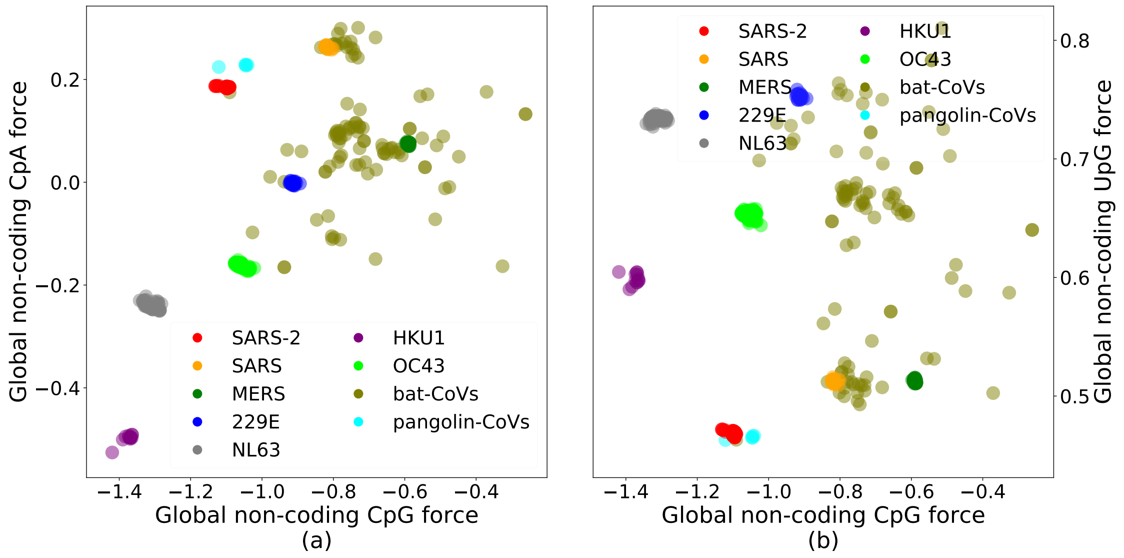

Figure SI.3: CpG versus CpA (left) and UpG (right) non-coding forces. Differently from the CpG versus UpA case (Fig. 2b), no clear correlations are found (r^2 equal to 0.2 and 0.01, respectively, for the left and right plots).

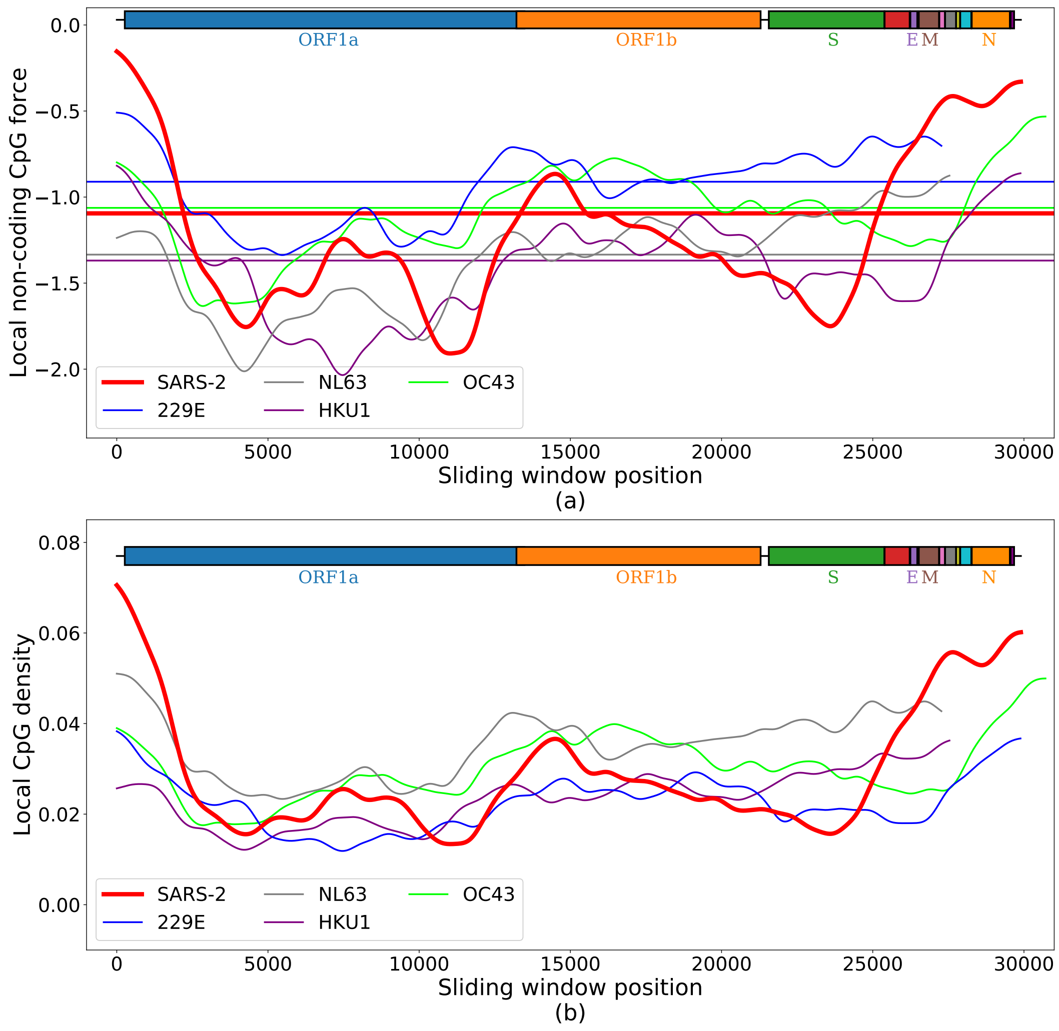

Figure SI.4: Supplement to Fig. 2 and Suppl. Fig. SI.2, where all the coronavirus species associated with circulating human strains are compared with SARS-CoV-2 in terms of CpG non-coding force (panel (a)) or number in fixed-length windows (panel (b)). Again, even though the final regions of the hCoVs has relatively high CpG force with respect to the other parts of their sequences, SARS-CoV-2 has a 3'-end CpG-force peak well above the final region of hCoV virus.

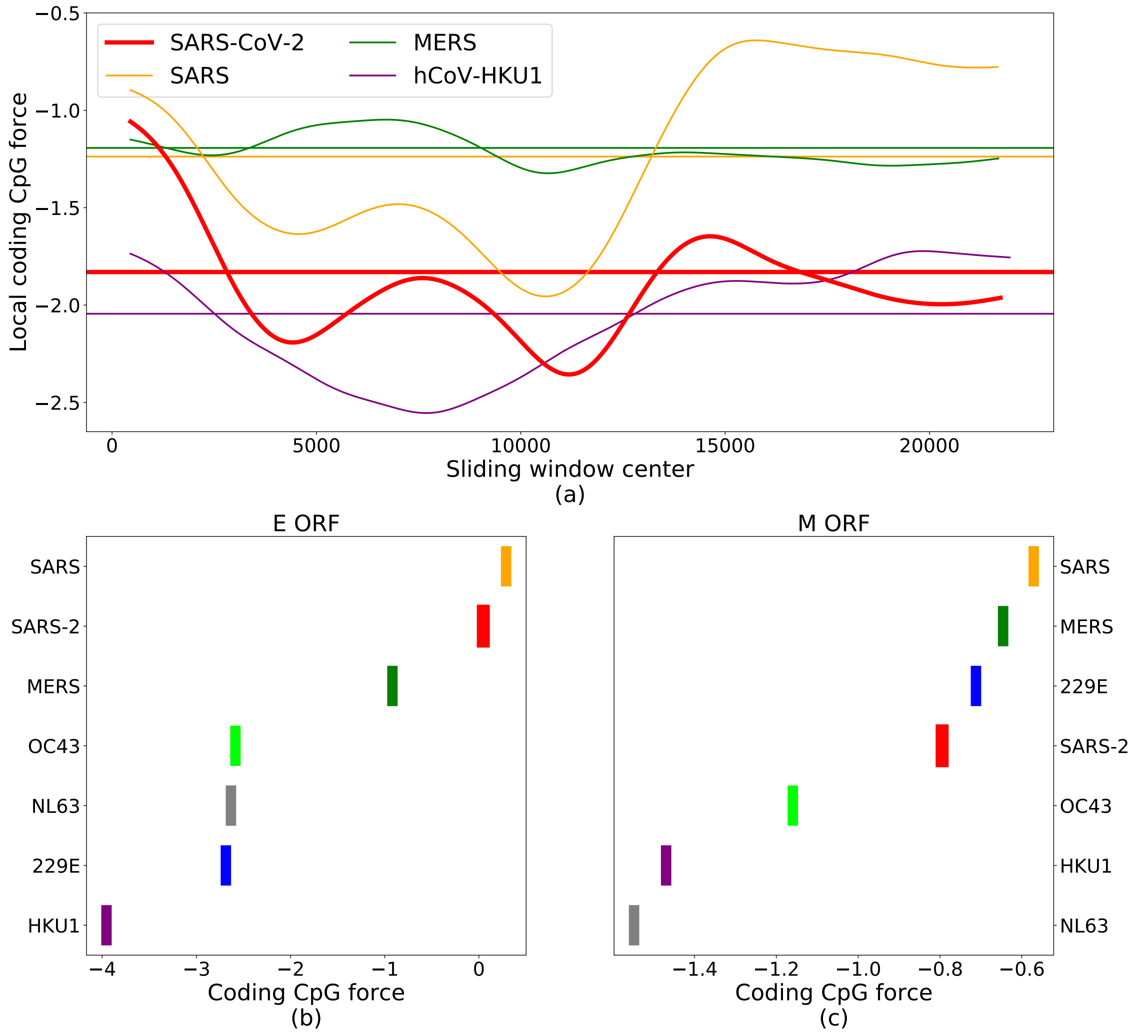

Figure SI.5: Extension of the comparison performed in Fig. 3. In panel (a) the CpG coding forces computed on the genome coding for polyprotein ORF1ab is compared among several coronaviruses and in panels (b) and (c) the regions coding for structural proteins E (envelope) and M (membrane) are considered. Notice that, due to the small size of proteins E and M, only one window is used so the boxes collapse in one line corresponding to the global CpG force on the protein, computed as an average over 4 to 20 viral genomes.

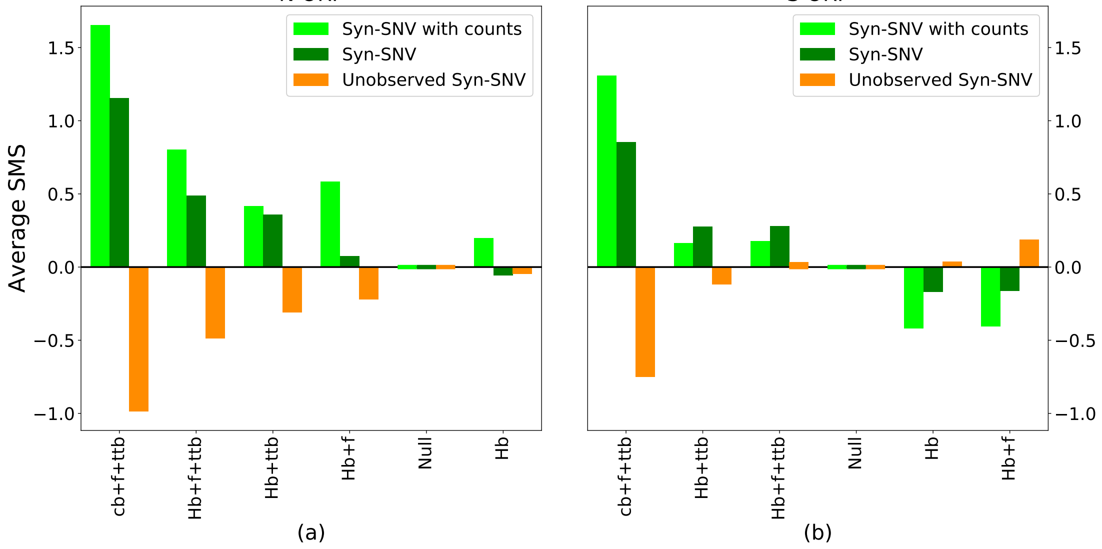

Figure SI.6: Supplementary information for Figs. 5e and 5f, where the model with human codon bias (Hb), with or without CpG force (f) and transition-transversion bias (ttb), is compared to the null model and the the model with virus codon bias (cb), CpG force and transition-transversion bias, in terms of average SMS assigned to synonymous SNV in N and S ORFs.

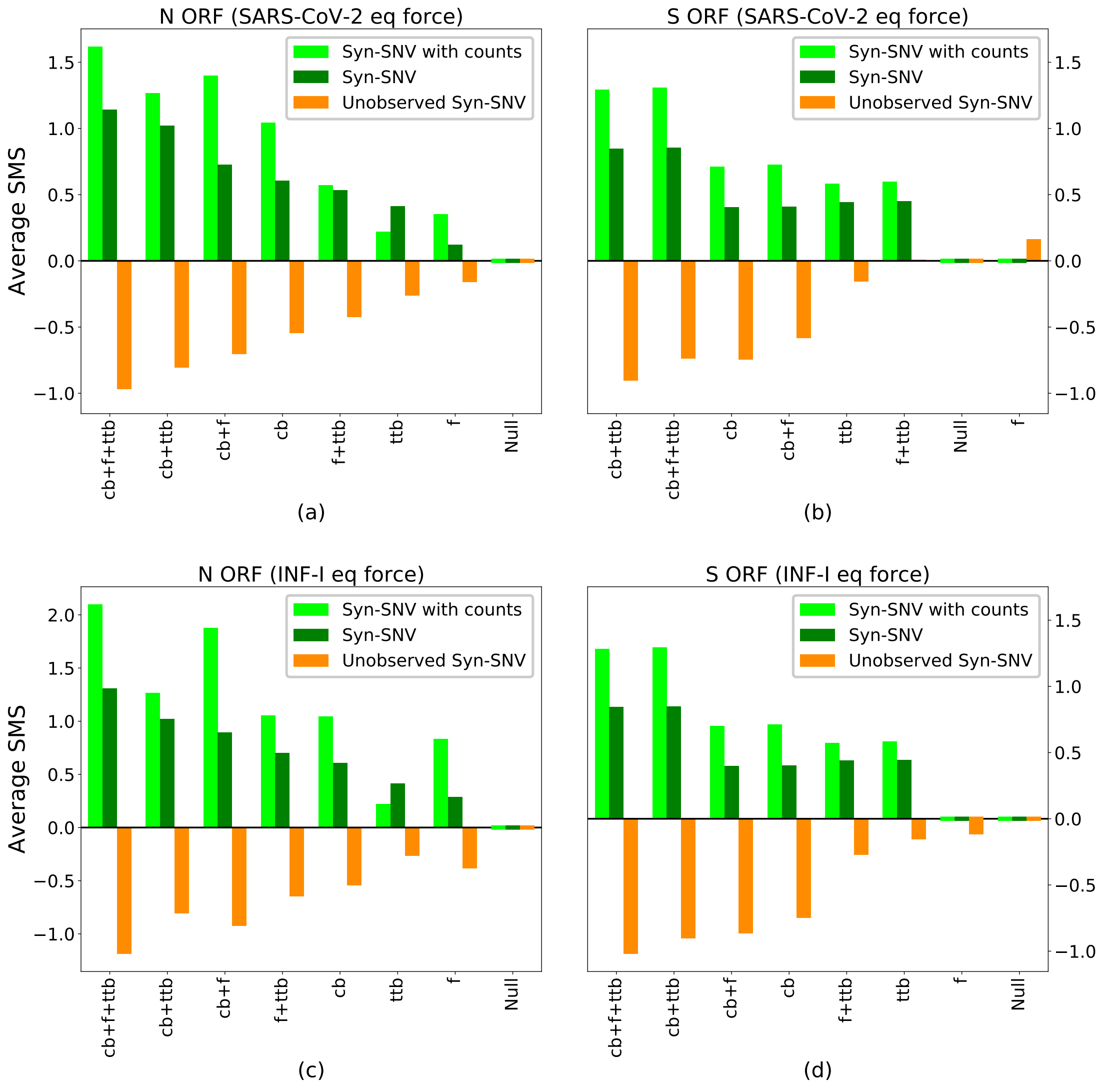

Figure SI.7: Same of Figs. 5e and 5f, for different choices of equilibrium force. (a), (b): the equilibrium force chosen is -1.71, that is the global coding force on SARS-CoV-2 (computed with the human codon bias as background). (c), (d): the equilibrium force chosen is -2.89, that is the average global non-coding force of human type-I interferon transcripts (computed with the coding human nucleotides as background).

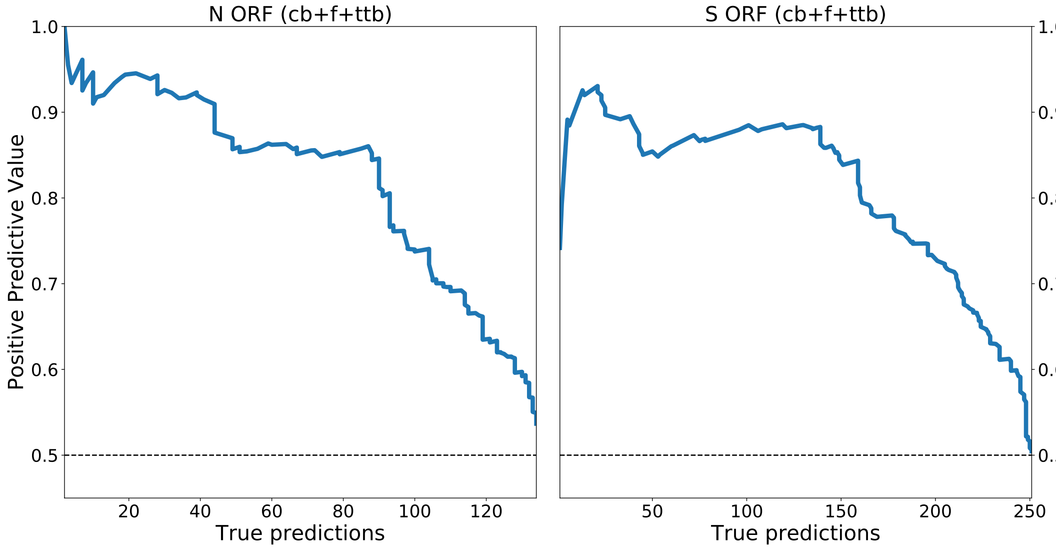

Figure SI.8: Positive Predictive Value for the model with virus codon bias, CpG force and transition-transversion bias, for N and S ORFs, as a function of the number of correctly predicted synonymous SNV.

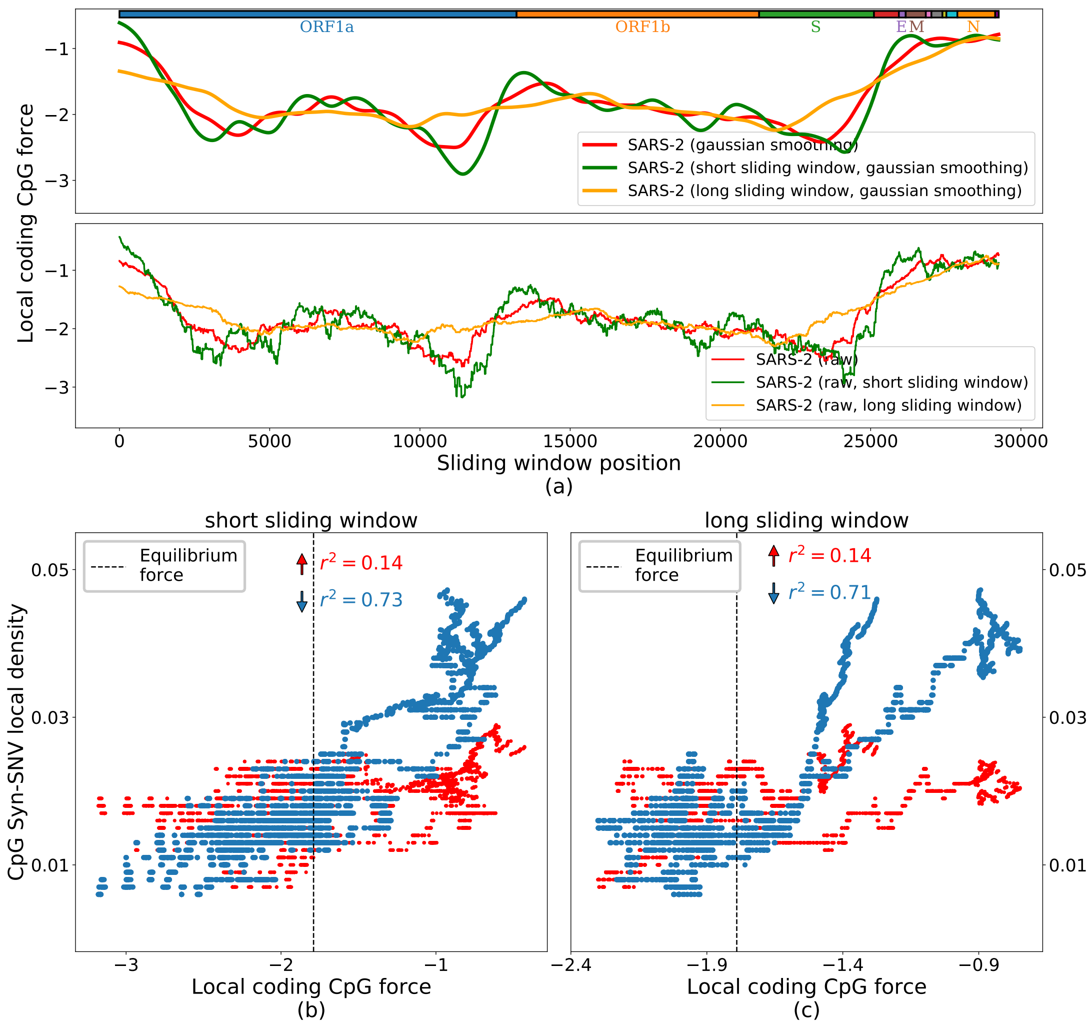

Figure SI.9: (a) Forces computed on SARS-CoV-2 ancestral genome, with different choices of sliding windows (upper subpanel), or without Gaussian smoothing (lower subpanel). The sliding windows used in panel (a) are: 3000 nt, 1500 nt (short sliding window), or 6000 nt (long sliding window). In panels (b), (c) we replicate the analysis performed for Fig. 4b with the short and long windows, to show that the results are qualitatively similar. The slightly lower performance of the short sliding window is due to the high sensitivity of the short windows for local details of the sequence, while the large window analysis is plagued by finite size effects (right side of panel (c)), due to the fact that the window size is similar to the total size of the sequence, together with the presence of many CpG motifs at the beginning and end of the sequence.

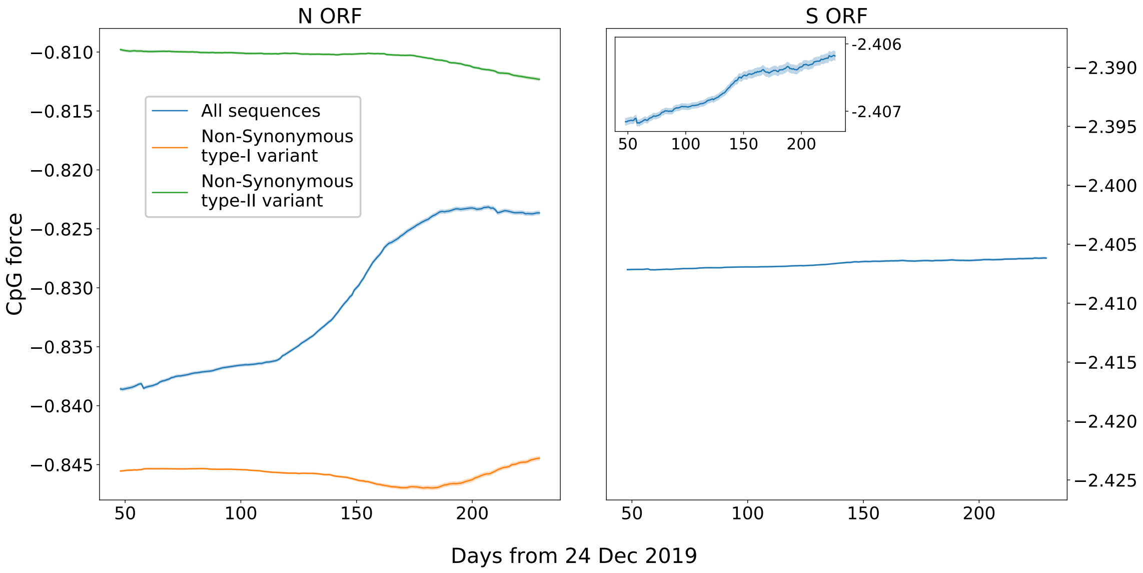

Figure SI.10: CpG local force evolution of N ORF and S ORF. Points are obtained by averaging the local CpG forces of the sequences collected in a temporal sliding window of 100 days centered on the point, shaded areas denote $\pm$ 1 standard deviation of this average. Differently from the direct analysis of CpG synonymous variants in Fig. 4c of the main text, it is difficult to disentangle synonymous mutations from the non-synonymous mutations in the evolution of the local force. This is shown here by comparing the local force evolution averaged on all the sampled sequences (48512 in number, blue curve) to the one averaged over sequences subdivided in the type-I variants (28456 in number, orange curve) which share the same codons in positions 202, 203 with the ancestral sequence (up to 1 SNV), and type-II variants (20054 in number, green curve) which presents 3 SNV (resulting in 2 amino acid substitutions and 1 extra CpG) and has been observed since 16 Feb 2020 in Europe. While the local force evolution averaged on all the sequences increases (such increases can be explained in terms of the ratio of number of sequences in the two variants) the local force average of type-II variants starts from a larger value (from the additional CpG) and slightly decreases in the last 50 days of this analysis. Force changes in S ORF are even less pronounced. The inset in the S ORF panel shows a zoom in the y axis of the S ORF CpG force evolution.

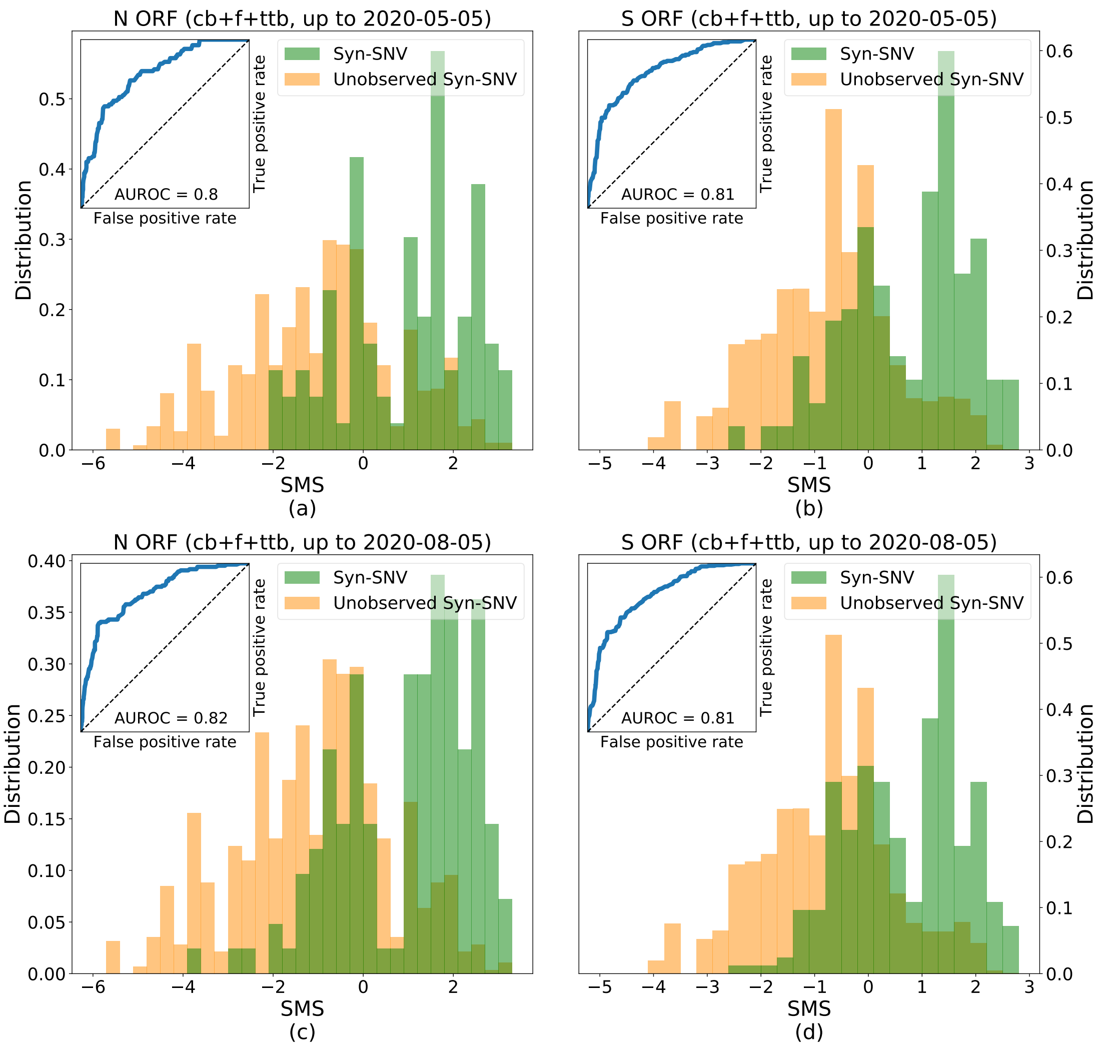

Figure SI.11: Same analysis performed in Figs. 5c and 5d, for sequences submitted to GISAID up to 5 May 2020 (panels (a), (b)) and up to 5 August 2020 (panels (c), (d)). The model cb+f+ttb can distinguish with large AUROCs (≥ 0.8), since 05 May 2020, observed Syn-SNV from conserved Syn-nt, and the model performance (intended here as AUROC) improves with time. Cutoffs for considering Syn-SNV are at 1 count for panels (a), (b), and at 2 counts for panels (c) and (d) (that is, approximately at 0.01% of the total number of collected sequences, as in main text for the full dataset). Other detailed analysis on sequences collected at previous times can be found in the previous versions of this manuscript on bioRxiv (https://www.biorxiv.org/content/10.1101/2020.05.06.074039v3.article-info).

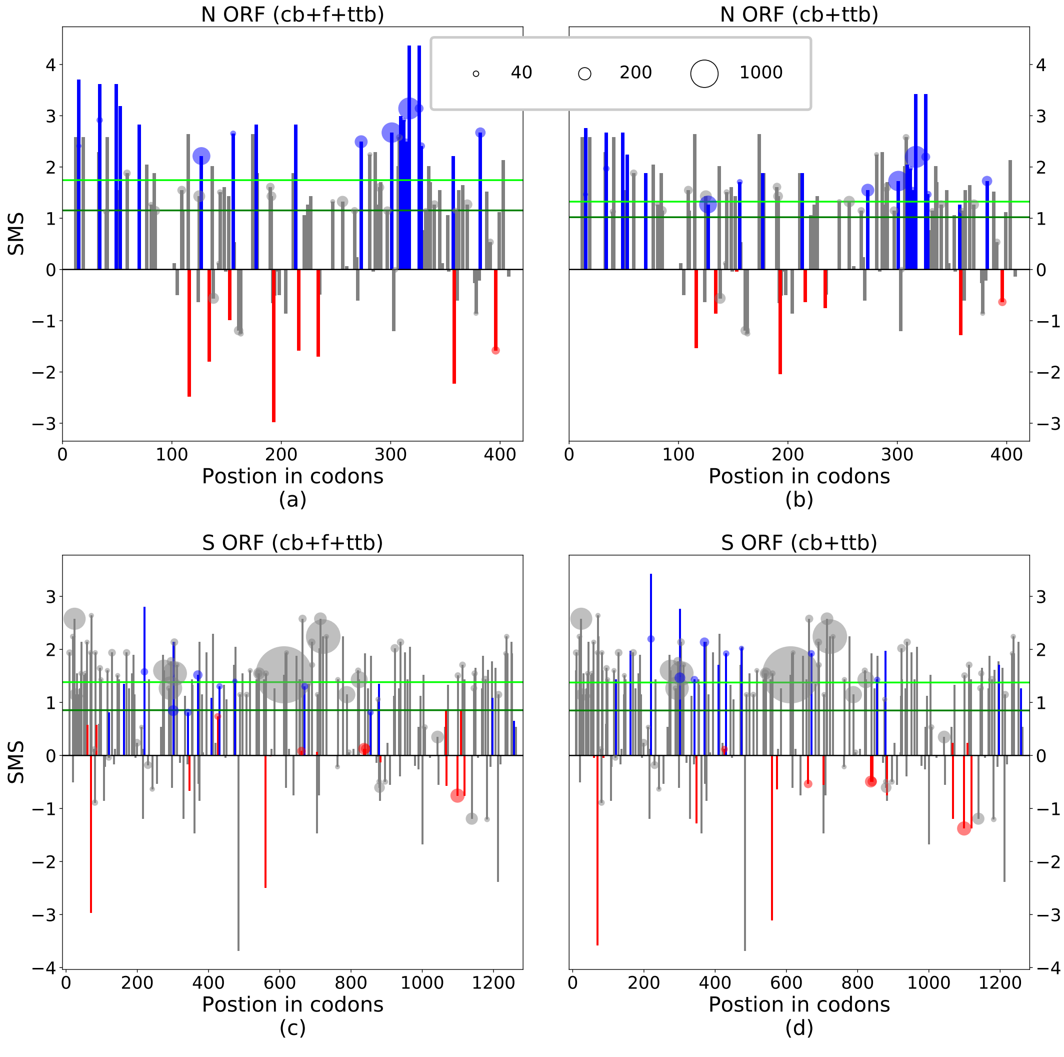

Figure SI.12: The SMS of all observed syn-SNV is given for N and S ORFs, with and without CpG drive. Bars colored in blue (red) correspond to CpG decreasing (increasing) syn-SNV. Circles on top of bars are drawn when the number of counts of the corresponding SNV is larger than 20, the size of the circle being proportional to the number of counts. Green (dark, light) horizontal lines give the average SMS (without or with counts).

Motif	n	Position of CpG	CpG↓ Syn-SNV with counts	ZAS
**C**GUGUUGCA**G**C**CG**	8	163	83	1.71
GGCUGUCACU**CG**	-	46	7	0.15
AGUGCACUCA**CG**	-	68	19	0.15
ACGAGUAACU**CG**	-	118	61	0.30
CUUACGGUUU**CG**	-	148	1122	0.30
CGGUUUCGUC**CG**	-	152	21	0.45
AUCUAGGUUU**CG**	-	186	46095	0.15
AGGUUUCGUC**CG**	-	190	31	0.30
CGGGUGUGAC**CG**	-	200	7	0.30
**C**UAAUUACU**G**U**CG**	8	96	0	1.56

Table SI.1: Analysis of CpG extended motifs of the form CnxGxCG with n = 4, 5, 6, 7 or 8 nucleotides in the 5'UTR. The position is given with respect to the start of the 5'UTR in the ancestral sequence, see Methods. All SNV happening in UTRs are considered synonymous. SNV observed with less than 5 counts are excluded from this analysis.

	CpGext					CpGext↓SNV									
						num					with counts				
n =	4	5	6	7	8	4	5	6	7	8	4	5	6	7	8
**5'UTR**	0	0	0	0	2	0	0	0	0	2	0	0	0	0	83
**N**	2	4	0	3	3	1	3	0	5	1	509	620	0	448	10
**M**	0	1	0	1	0	0	0	0	0	0	0	0	0	0	0
**ORF7a**	1	0	0	1	0	0	0	0	1	0	0	0	17	0	0
**ORF3a**	1	0	1	1	1	0	0	0	0	0	0	0	0	0	0
**ORF1a**	2	7	5	7	4	1	5	1	6	2	7	230	8	338	143
**ORF1b**	3	2	5	4	2	2	1	4	1	1	19	113	68	98	19
**S**	0	1	2	0	0	0	0	1	0	0	0	0	25	0	0

Table SI.2: Supplementary information for Table 5. Only motifs of the form CnxGxCG with n = 4, 5, 6, 7 or 8 (CpGext) are considered and they are only characterized by the spacer length n. The number of CpGext motifs and of syn-SNV removing them is given for each value of n for each ORF or UTR (with at least 1 CpGext).

	Average SMS				ANOVA F-test	
	SNV	u-SNV	diff	AUROC	F	num items
**3'UTR**	0.33	-0.39	0.73	0.77	72.2	81 + 405
**5'UTR**	0.57	-0.34	0.91	0.83	81.9	56 + 577
**N**	1.02	-0.81	1.83	0.83	162.0	114 + 977
**M**	1.12	-0.70	1.81	0.85	94.5	58 + 557
ORF10	1.37	-0.51	1.89	0.84	9.8	6 + 87
**ORF7a**	0.30	-0.77	1.08	0.71	12.4	23 + 289
**ORF8**	0.77	-0.84	1.62	0.83	36.8	25 + 266
**ORF3a**	1.06	-0.80	1.86	0.85	96.9	54 + 642
**ORF1a**	0.93	-0.89	1.83	0.86	1580.3	848 + 9966
**ORF1b**	0.78	-0.87	1.63	0.82	624.9	432 + 6026
E	0.35	-0.56	0.91	0.68	3.6	10 + 217
**S**	0.85	-0.90	1.75	0.84	375.2	223 + 2924
ORF6	0.35	-0.56	0.91	0.70	5.3	10 + 127
ORF7b	0.70	-0.41	1.11	0.77	6.1	9 + 98

Table SI.3: Supplementary information for Table 6, where all the quantities are computed through the model cb+ttb (without CpG drive). The regions in bold are the most reliable for statistical analysis as they present at least 20 syn-SNV (see Table 3).

	ave SMS diff		AUROC	
model:	cb+ttb	cb+f+ttb	cb+ttb	cb+f+ttb
**3'UTR**	0.75	1.08	0.79	0.84
**5'UTR**	1.54	3.18	0.97	0.99
**N**	2.13	2.73	0.88	0.89
**M**	0.95	1.08	0.68	0.68
ORF10	1.44	1.67	0.79	0.76
**ORF7a**	1.75	2.10	0.84	0.85
**ORF8**	1.72	1.89	0.85	0.85
**ORF3a**	2.19	2.38	0.91	0.91
**ORF1a**	2.17	2.16	0.91	0.91
**ORF1b**	1.92	1.92	0.90	0.90
E	1.10	1.59	0.76	0.76
**S**	2.28	2.14	0.91	0.92
ORF6	0.72	0.73	0.69	0.69
ORF7b	1.29	1.30	0.84	0.84

Table SI.4: Supplementary information for Table 6, where ave SMS diff and AUROC are given for the models cb+ttb and cb+f+ttb while taking into account syn-SNV counts for the computation. In both cases, counts are used to weight observed syn-SNV. Notice that taking counts into account can lead to strong bias effects if few mutations are observed extremely more often than all others. For instance, in ORF1a the mutation observed more often has alone more than 50% of the total number of counts for the ORF, hence it strongly influences all indicators in this table. Similar effects are seen for 5'UTR, M, and ORF8.

SI.3 From CpG force to CpG relative abundance

We want to show in which limit the CpG force (without codon constraints) is equivalent to the relative dinucleotide abundance [2], Eq. (4). We start from the partition function:

$$Z = \sum_{s_1,\ldots,s_N} \left(\prod_{i=1}^{N} f(s_i) \right) \prod_{i=1}^{N-1} e^{x\delta(s_i,a)\delta(s_{i+1},b)}, \tag{SI.1}$$

where δ denotes the Kroneker delta function. In the spirit of a cluster expansion, we write

$$e^{x\delta(s_i,a)\delta(s_{i+1},b)} = 1 + g_{i,i+1}, \tag{SI.2}$$

where

$$g_{i,i+1} = (e^x - 1)\,\delta(s_i, a)\,\delta(s_{i+1}, b). \tag{SI.3}$$

Inserting back this into Eq. (SI.1), we obtain

$$\begin{aligned} Z &= \sum_{s_1,\ldots,s_N} \left(\prod_{i=1}^{N} f(s_i) \right) \prod_{i=1}^{N-1} (1 + g_{i,i+1}) \\ &= \sum_{s_1,\ldots,s_N} \left(\prod_{i=1}^{N} f(s_i) \right) \left[1 + \sum_i g_{i,i+1} + \sum_{i<j} g_{i,i+1}\, g_{j,j+1} + \ldots \right]. \end{aligned} \tag{SI.4}$$

Now we can compute each term in the cluster expansion, and we get for the k-th term (for $a \neq b$, as in the CpG case)

$$\sum_{s_1,\ldots,s_N} \left(\prod_{i=1}^{N} f(s_i) \right) \sum_{i_1<\cdots<i_k} g_{i_1,i_1+1} \cdots g_{i_k,i_k+1} = \binom{N-k}{k} \left((e^x - 1)\, f(a)\, f(b) \right)^k = \binom{N-k}{k} g^k. \tag{SI.5}$$

where we defined $g = (e^x - 1)\, f(a)\, f(b)$. Now we suppose $N = 2m$, that is N is even (however, we will consider soon the large-N limit, where this request is not necessary anymore). Therefore, we have

$$Z = \sum_{k=0}^{m} \binom{2m-k}{k} g^k = \frac{(1 + 2g - \sqrt{1+4g})^m (\sqrt{1+4g} - 1) + (1 + 2g + \sqrt{1+4g})^m (\sqrt{1+4g} + 1)}{2^{m+1}\sqrt{1+4g}}. \tag{SI.6}$$

To proceed further, we can consider the case where $g \ll 1$. This is a good approximation when $x \simeq 0$, and it is also fairly good as long as x is lower than 0, but it is less good for the most negative forces observed here (see Fig. 3a). Under this hypothesis, we have

$$Z = (1 + g)\, e^{(m-1)2g} \simeq e^{N(e^x - 1)\, f(a)\, f(b)}, \tag{SI.7}$$

where in the last step we used also that $N \gg 1$. From this, by using that $\langle n \rangle = \partial_x \log Z$ and requesting $\langle n \rangle = n_0 = N f(ab)$, we obtain Eq. (4). Fig. SI.13 shows the correlation between the CpG force with the nucleotide bias and the CpG relative abundance.

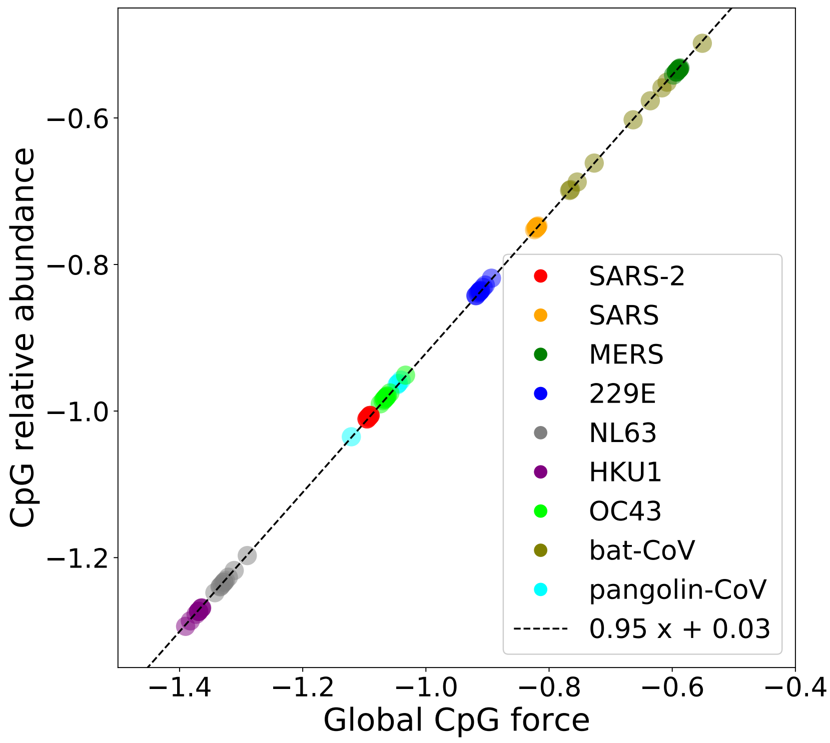

Figure SI.13: Comparison between the CpG force and the CpG relative abundance index. As discussed in Sec. SI.3, these two quantities are almost identical when the genome is long and the force is small in absolute value. Here 10 different genomes for several coronavirus species are used to compute these two quantities, and the dashed black line is a linear fit of the resulting points.

References

[1] Kristian G Andersen, Andrew Rambaut, W Ian Lipkin, Edward C Holmes, and Robert F Garry. The proximal origin of sars-cov-2. *Nature medicine*, 26(4):450–452, 2020. 1

[2] Samuel Karlin and Jan Mrázek. Compositional differences within and between eukaryotic genomes. *Proceedings of the National Academy of Sciences*, 94(19):10227–10232, 1997. 12
